# Mitochondrial proteomics of nasopharyngeal carcinoma metastasis

**DOI:** 10.1186/1755-8794-5-62

**Published:** 2012-12-06

**Authors:** Jianping Liu, Xianquan Zhan, Maoyu Li, Guoqing Li, Pengfei Zhang, Zhefeng Xiao, Meiying Shao, Fang Peng, Rong Hu, Zhuchu Chen

**Affiliations:** 1 Key Laboratory of Cancer Proteomics of Chinese Ministry of Health, Xiangya Hospital, Central South University, 87 Xiangya Road, Changsha, Hunan, 410008, P.R. China; 2 Hunan Engineering Laboratory for Structural Biology and Drug Design, Xiangya Hospital, Central South University, 87 Xiangya Road, Changsha, Hunan, 410008, P.R. China; 3 Cancer Research Institute, Xiangya School of Medicine, Central South University, 88 Xiangya Road, Changsha, Hunan, 410078, P.R. China; 4 Bio-Analytical Chemistry Research Laboratory, Modern Analytical Testing Center, Central South University, 88 Xiangya Road, Changsha, Hunan, 410078, P.R. China; 5 Department of Biology, School of Pharmacy and Life Science, University of South China, Hengyang, 421001, P.R. China

**Keywords:** Nasopharyngeal carcinoma, Tumor metastasis, Mitochondria, Proteome, Differentially expressed proteins, Functional enrichment analysis, Literature data mining, PRDX3

## Abstract

**Background:**

Mitochondrial proteomic alterations of nasopharyngeal carcinoma metastasis remain unknown. Our purpose is to screen mitochondrial proteins for the elucidation of the molecular mechanisms of nasopharyngeal carcinoma metastasis and the discovery of metastasis-related biomarkers.

**Methods:**

Mitochondria were isolated from nasopharyngeal carcinoma metastatic (5-8F) and nonmetastatic (6-10B) cell lines, respectively. After characterization of isolated mitochondria, mitochondrial differentially expressed proteins (DEPs) were quantified by two-dimensional difference in-gel electrophoresis (2D-DIGE), and identified by peptide mass fingerprint (PMF) and tandem mass spectrometry (MS/MS). A functional enrichment analysis and a protein-protein interaction sub-network analysis for DEPs were carried out with bioinformatics. Furthermore, siRNAs transient transfections were used to suppress expressions of some up-regulated DEPs in metastatic cells (5-8F), followed by Transwell Migration assay.

**Results:**

Sixteen mitochondrial DEPs including PRDX3 and SOD2 were identified. Those 5-8F cells with suppression of PRDX3 showed an increased mobility potential. The functional enrichment analyses of DEPs discovered five significant biological processes including cellular response to reactive oxygen species, hydrogen peroxide metabolic process, regulation of mitochondrial membrane potential, cell redox homeostasis and oxidation reduction, and five significant molecular functions including oxidoreductase activity, caspase inhibitor activity, peroxiredoxin activity, porin activity and antioxidant activity. A protein-protein interaction sub-network of DEPs was generated with literature data. Ten mitochondrial DEPs including PRDX3, PRDX6, SOD2, ECH1, SERPINB5, COX5A, PDIA5, EIF5A, IDH3B, and PSMC4 were rationalized in the tumor-stroma co-evolution model that mitochondrial oxidative stress directly contributes to tumor metastasis.

**Conclusions:**

Sixteen mitochondrial DEPs were identified with mass spectrometry and ten of them were rationalized in the tumor-stroma co-evolution model. Those 5-8F cells with suppression of PRDX3 showed an increased mobility potential. These data suggest that those mitochondrial DEPs are potential biomarkers for NPC metastasis, and their dysregulation would play important roles in mitochondria oxidative stress-mediated NPC metastatic process.

## Background

Nasopharyngeal carcinoma (NPC) cell lines 5-8F and 6-10B are derived from the same human cell line SUNE1 that is derived from a female poorly differentiated nasopharyngeal squamous epithelial cell carcinoma. Those two cell sublines 5-8F and 6-10B with the same genetic background have tumorigenic ability that was confirmed by the colony formation in soft agar and in vivo mouse model, but are quite different in metastatic potential [1-3]. 5-8F cells are metastatic and 6-10B cells are nonmetastatic, which was confirmed by in vivo mouse model [2]. This different biological character shows that these two cell sublines are the useful experiment materials to study the molecular events of NPC metastasis. Based on those two subcelines, some research groups analyzed the differentially expressed genes (DEGs) and differentially expressed proteins (DEPs) using the whole-cell samples of those two cell lines. A total of 78 DEGs were identified with subtractive suppression hybridization [4] and microarray [5]. Of them, 11 DEGs including PTHLH formed a metastasis-related gene network [5]. A total of 28 DEPs were identified with two-dimensional gel electrophoresis (2DGE) coupled with matrix-assisted laser desorption ionization time-of-flight mass spectrometry (MALDI-TOF MS) [6,7]. Those DEPs were related to apoptosis, cell cycle, cell signal transduction, cell survival, transcription regulation, cell mobility, protein synthesis, and DNA damage repair [6,7]. However, those DEGs and DEPs were not fully rationalized in the biological process of NPC metastasis, and there is no any overlap between those 78 DEGs and 28 DEPs [4-7]. It would be derived from the following limitation factors: (i) the range of the protein abundance of the whole-cell sample is too wide, not all proteins were extracted; (ii) 2DGE only can array very small partial components of a whole-cell proteome, and (iii) the process of translation of DEG to DEP is regulated by multiple factors, which results in the low consistence between the DEG and DEP profiles. The subcellular proteomics such as just focusing on one compartment of a cell would more effectively discover function-related proteins because the subcellular proteome is much simpler than the whole-cell proteome, it can maximally identify the protein components in a subcellular proteome.

Many evidences have demonstrated that mitochondria is the central to oxidative stress, and tumor cell self has anti-oxidant ability for its survival and metastasis [8-15]. Mitochondria are not just innocent bystander in cancer, but are related to tumorigenesis, apoptosis, cancer therapy and metastasis. The contribution of mitochondria to cancer involves three critical processes including alterations in glucose metabolism, production of reactive oxygen species (ROS), and compromise of intrinsic apoptotic function [8,9]. According to the tumor-stroma co-evolution model opinion, cancer cells secrete hydrogen peroxide, induce oxidative stress in adjacent stromal cells, and result in an excess production of ROS in mitochondria. Excess stromal ROS induces cytoskeleton rearrangements, autophagy, and mitophagy in the tumor microenvironment to help cancer cells escape excess ROS in the primary tumor site, also induces the stromal over-production of recycled nutrients such as ketones and L-lactate that are fuel for tumor growth and metastasis [10-14]. This model provides fresh insight of how mitochondria involved in tumor metastasis. Therefore, a tight relationship exists between mitochondria and tumor metastasis. However, the causes, consequences, and other series of related questions regarding molecular mechanism of mictochondria in tumor metastasis still remain unclear. Thus, this current work focused on the mitochondrial proteome difference between metastatic 5-8F and 6-10B cells, and provided clues to clarification of the functional role of mitochondria in the process of NPC metastasis.

In order to screen mitochondrial proteins for the elucidation of the mechanism of NPC metastasis and the discovery of metastasis-related biomarkers, two-dimensional difference in-gel electrophoresis (2D-DIGE) was used to determine mitochondrial DEPs between metastatic 5-8F and nonmetastastic 6-10B cells, followed by protein characterization with peptide mass fingerprint (PMF) and tandem mass spectrometry (MS/MS). siRNA transient transfection was used to suppress the expressions of up-regulated DEPs in metastatic 5-8F cells, followed by the observation of changes in cell mobility potential by Transwell Migration assay. Cytoscape BiNGO plugin was used for gene ontology (GO) analyses of DEPs to identify the enriched biological processes and molecular functions. A protein-protein interaction sub-network of DEPs was generated with literature data mining from HPRD and NCBI databases. Ten DEPs were rationalized in a tumor-stroma co-evolution model, and associated with tumor metastasis.

## Results

### Characterization of isolated mitochondria

**Figure 1:**
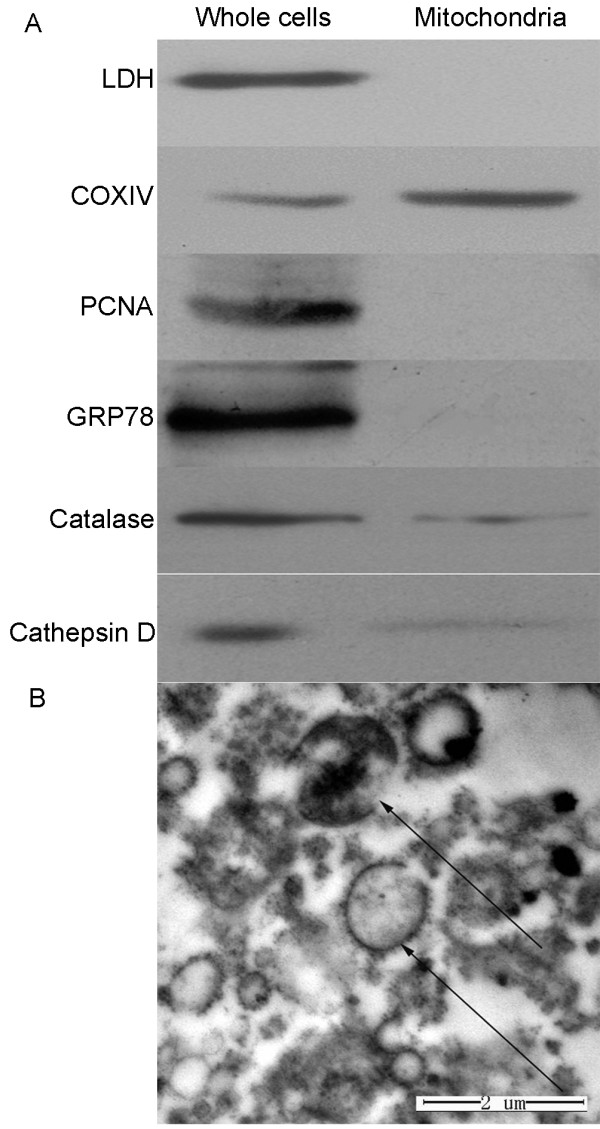
**Characterization of isolated mitochondria from cells. A**. Western blotting images of 6 marker proteins in the whole-cell protein samples (left column) and the isolated mitochondrial protein samples (right column). A total of 60 μg proteins was loaded and isolated by 12% SDS-PAGE, and transferred to PVDF membrane and incubated with primary antibodies (dilution 1:500) of each marker protein and second antibodies (dilution 1:2,000). **B**. Electron micrograph of isolated mitochondrial samples from cells (20,000 ×).

The purity of isolated mitochondria was assessed by Western blotting and electron microscopic observations. Figure 1A showed Western blotting images of extracted proteins from isolated mitochondrial samples against known marker proteins from cytoplasm (LDH), mitochondria (COX IV), nucleus (PCNA), endoplasmic reticulum (GRP 78), peroxisome (catalase), and lysosome (Cathepsin D). Mitochondrial protein COX IV was specifically detected in isolated mitochondrial sample and this sample lacked any detectable contamination of abundant cytosolic (LDH), nuclear (PCNA), and endoplasmic reticulum proteins (GRP78), except for detectable contamination of catalase from peroxisome and cathepsin D from lysosome. Figure 1B showed electron microscopic observation of isolated mitochondrial samples, where mitochondria had typical membrane architecture. All the results showed that isolated mitochondrial samples were full of intact mitochondria, though little contaminated by other sub-cellular compartments. 

### Detection of mitochondrial DEPs between metastatic and nonmetastatic cells by 2D-DIGE

**Figure 2:**
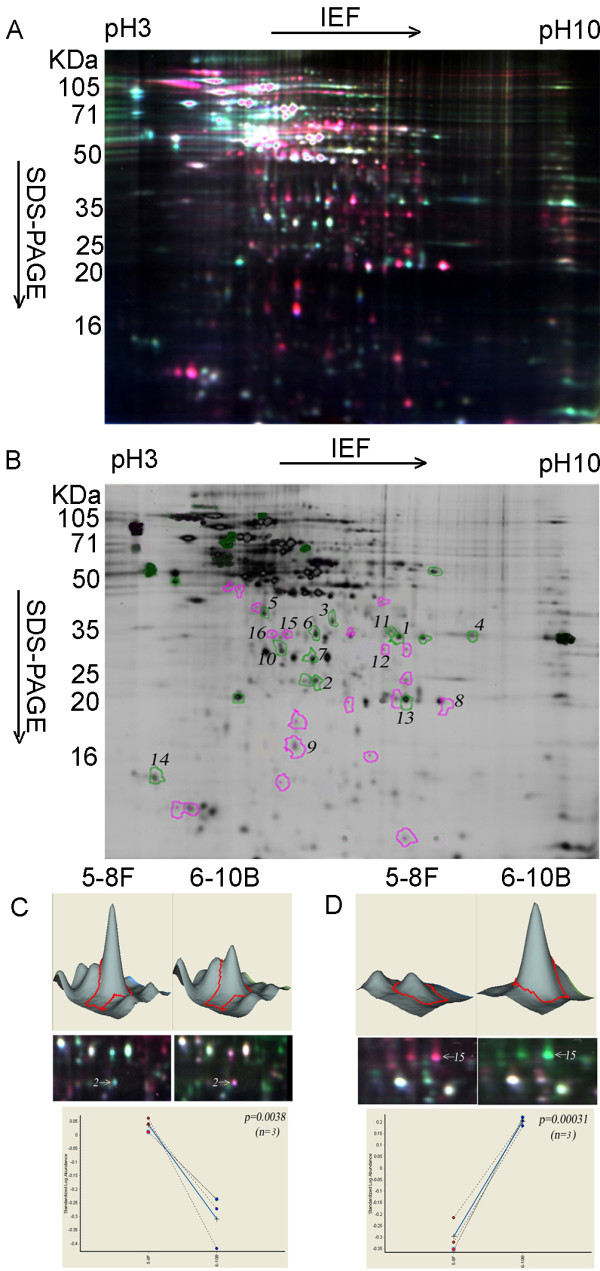
**2D-DIGE analysis of metastatic 5-8F versus nonmetastatic 6-10B cells. A**. A representative two-color merged 2D-DIGE gel image. Green is the Cy3-labeled metastatic 5-8F cell sample. Red is the Cy5-labeled 6-10B cell sample. **B**. Labeled differentially expressed proteins (DEPs) between metastatic (5-8F) and nonmetastatic (6-10B) cell mitochondria (Ratio = 5-8F/6-10B; Red: down-regulated; Green: up-regulated). **C**. Three-dimensional simulation, a close-up of the region of 2D-DIGE gel image, and the associated graph view of a representative up-regulated protein in metastatic 5-8F cell mitochondria (spot 2). D. Three-dimensional simulation, a close-up of the region of 2D-DIGE gel image, and the associated graph view of a representative down-regulated protein in metastatic 5-8F cell mitochondria (Spot 15).

Mitochondrial protein expressions were compared between metastatic 5-8F and nonmetastatic 6-10B cells using 2D-DIGE with a mixed-sample internal standard. After comparative analyses by DeCyder 5.0 software in all 9 protein-spot image maps, average of 1,047 ± 78 protein-spots were detected, in total 36 spots containing DEPs (X-fold_5-8F/6-10B_ > 2, P < 0.05) were detected. Figure 2A showed a representative two-color merged gel image. Figure 2B showed 36 spots containing DEPs. Figure 2C showed the three-dimensional simulation, a close-up of the region of 2D-DIGE gel image, and the associated graph view of an up-regulated protein (spot 2). Figure 2D showed the three-dimensional simulation, a close-up of the region of 2D-DIGE gel image, and the associated graph view of a down-regulated protein (spot 15). 

### Identification of mitochondrial DEPs between metastatic and nonmetastatic cells by PMF and MS/MS

All of 36 gel-spots that contained DEPs were excised from the stained preparative gel. The proteins in each excised gel spot were subjected to in situ digestion with trypsin, and analyzed with MALDI-TOF PMF and ESI-Q-TOF MS/MS. All PMF-based identification data were collected in the supplementary mass spectral data (Additional file 1: Figures 1-13). All MS/MS-based identification data were collected in the supplementary mass spectral data (Additional file 1: Figures 14-19). A total of 16 DEPs were identified, including 4 down-regulated proteins (IDH3B, COX5A, SERPINB5, and MRPL12) and 12 up-regulated proteins (PRDX3, PRDX6, SOD2, PDIA5, ETFA, VDAC1, VDAC2, ECH1, PHB, EIF5A, PSMC4, and CALU) (Table 1). Those DEPs function in mitochondria ATP synthesis, ion transport, hydrogen peroxide metabolic process, DNA damage response, apoptosis, cell redox homeostasis, respiratory electron transport, fatty acid beta-oxidation, regulation of transcription, protein biosynthesis, and mitochondrial membrane potential. 

**Table 1:** Differentially expressed proteins in mitochondria of metastatic 5-8F compared to non-metastatic 6-10B cells, identified by 2D-DIGE and MS (Ratio = 5-8F/6-10B; +, up-regulated; -, down-regulated)

**Proteins names**	**Spots no.**	**Accession number**	**Gene names**	**Subcellular location**	**Mass (Da)**	**pI**	**Mascot score**	**NMP**	**NUP**	**MOI**	**Sequence coverage (%)**	**Average ratio**
1. Oxidoreductase Proteins												
Peroxiredoxin 3	2	gi|119569781	PRDX3	Mitochondria	24,941	5.73	90	5	9	PMF	43	+2.2
Peroxiredoxin 6	10	gi|4758638	PRDX6	Mitochondria, lysosome	25,133	8.36	71	5	8	PMF	21	+2.5
Superoxide dismutase	13	gi|67782309	SOD2	Mitochondria	20,882	8.36	71	4	4	PMF	52	+3.3
[Mn], mitochondrial isoform B precursor							62	2	\	MS/MS	8	
Isocitrate dehydrogenase	12	gi|5031777	IDH3B	Mitochondria	40,022	6.47	75	6	10	PMF	15	-3.3
[NAD] subunit alpha, mitochondrial precursor							73	1	\	MS/MS	3	
Protein disulfide isomerase related protein 5	5	gi|1710248	PDIA5	Endoplasmic reticulum	46,512	4.95	166	9	4	PMF	37	+2.4
Cytochrome c oxidase subunit 5A, mitochondrial precursor	9	gi|190885499	COX5A	Mitochondria	16,923	6.30	72	4	3	PMF	38	-4.4
Electron transfer	6	gi|2781202	ETFA	Mitochondria	33,418	6.95	73	4	2	PMF	27	+2.6
flavoprotein							95	3	\	MS/MS	11	
2. Porin Proteins												
Volatage-dependent anion selective channel protein 1	4	gi|4507879	VDAC1	Mitochondria	30,868	8.62	117	7	8	PMF	38	+2.4
Volatage-dependent anion channel 2	1	gi|55664661	VDAC2	Mitochondria	30,842	8.00	101	6	5	PMF	34	+2.2
3. Metabolism and regulation Proteins												
Delta(3.5)-Delta(2.4)-dienovl-CoA isomerase, mitochondrial precursor	11	gi|70995211	ECH1	Mitochondria	36,136	8.16	105	7	10	PMF	44	+4.1
Prohibitin	7	gi|4505773	PHB	Mitochondria	29.843	5.57	158	8	1	PMF	44	+2.0
Maspin	15	gi|56554671	SERPINB5	Extracellular region	43,690	5.99	225	5	\	MS/MS	15	-3.2
Eukaryotic initiation factor 5A	14	gi|4261795	EIF5A	Endoplasmic reticulum	17,018	4.85	68	3	\	MS/MS	15	+3.2
26S protease regulatory subunit 7 isoform 1	3	gi|4506209	PSMC4	Mitochondria, proteasome	49,002	5.71	92	6	2	PMF	19	+2.0
Ribosomal protein L7/L12, mitochondria	8	gi|1313962	MRPL12	Mitochindria	21,593	9.04	86	4	\	MS/MS	19	-3.9
Calumenin isoform c precursor	16	gi|314122177	CALU	Endoplasmic reticulum	38,141	4.47	124	8	9	PMF	31	+2.9

NMP, the number of matched peptides. NUP, the number of unmatched peptides. MOI, methods of identification.

### Functional enrichment analyses of mitochondrial DEPs and protein-protein interaction data

**Figure 3:**
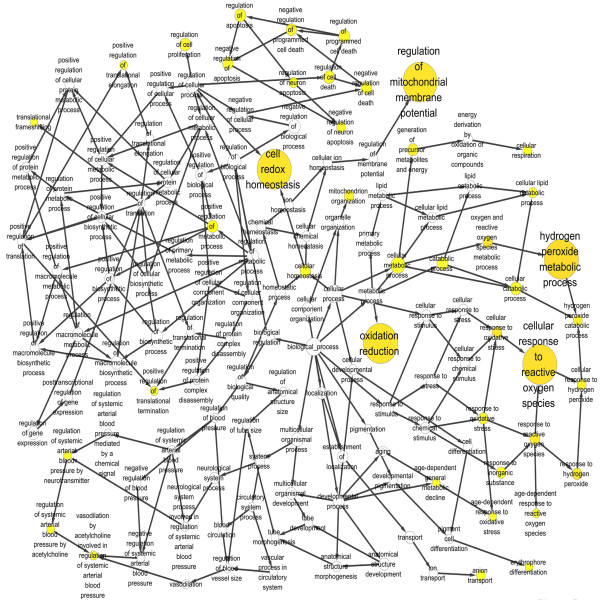
**Enriched biological processes of differentially expressed mitochondrial proteins between metastatic 5-8F and nonmetastatic 6-10B cells.** A circle represents a biological process. An arrow represents the relationship between two biological processes. The color from white to deep yellow represents different p-values.

**Figure 4:**
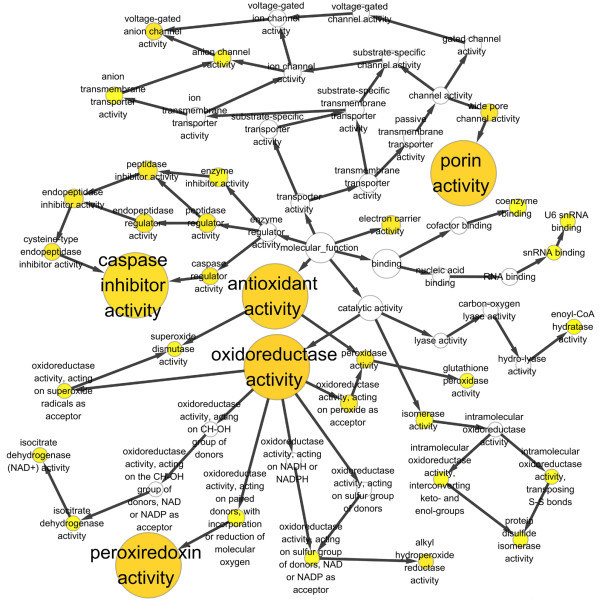
**Enriched molecular functions of differentially expressed mitochondrial proteins between metastatic 5-8F and nonmetastatic 6-10B cells.** A circle represents a molecular function. An arrow represents the relationship between two molecular functions. The color from white to deep yellow represents different p-values.

Functional enrichment categories that characterized mitochondrial DEPs were identified. The functional enrichment maps of biological process (Figure 3) and molecular function (Figure 4) were shown. The statistical significantly enriched biological processes and molecular functions (p < 0.05) were listed in Table 2. Biological processes such as cellular response to reactive oxygen species, hydrogen peroxide metabolic process, regulation of mitochondrial membrane potential, cell redox homestasis, and oxidation reduction were enriched. Molecular functions such as oxidoreductase activity, caspase inhibitor activity, peroxiredoxin activity, porin activity, and antioxidant activity were enriched. These results obviously showed that chiefly enriched proteins for the identified mitochondrial DEPs were oxidoreducition-related proteins and chiefly enriched biological processes were oxidoreducition-related processes. 

**Table 2:** Enriched Go terms of differentially expressed proteins in mitochondria of metastatic 5-8F relative to non-metastatic 6-10B cells

**GO-ID**	**Description**	**P-Value**	**Genes**
Biological Process			
34614	Cellular response to reactive oxygen species	7.09E-6	PRDX6↑, PRDX3↑, SOD2↑
42743	Hydrogen peroxide metabolic process	2.39E-6	PRDX6↑, PRDX3↑, SOD2↑
51881	Regulation of mitochondria membrane potential	5.62E-5	PRDX3↑, SOD2↑
45454	Cell redox homeostasis	3.92E-5	PRDX6↑, PDIA5↑, PRDX3↑
55114	Oxidation reduction	2.92E-5	PRDX6↑, IDH3B↓, PDIA5↑, PRDX3↑, ETFA↑, SOD2↑
Molecular Function			
16491	Oxidoreductase activity	2.64E-6	PRDX6↑, IDH3B↓, PDIA5↑, PRDX3↑, COX5A↓, ETFA ↑, SOD2↑
43027	Caspase inhibitor activity	6.61E-5	PRDX6↑, PRDX3↑
51920	Peroxiredoxin activity	1.51E-5	PRDX6↑, PRDX3↑
15288	Porin activity	1.51E-5	VDAC2↑, VDAC1↑
16209	Antioxidant activity	1.35E-5	PRDX6↑, PRDX3↑, SOD2↑

↑ : up- regulated expression in metastatic 5-8F cells relative to non-metastatic 6-10B cells.

↓ : down-regulated expression in metastatic 5-8F cells relative to non-metastaic 6-10B cells.

**Figure 5:**
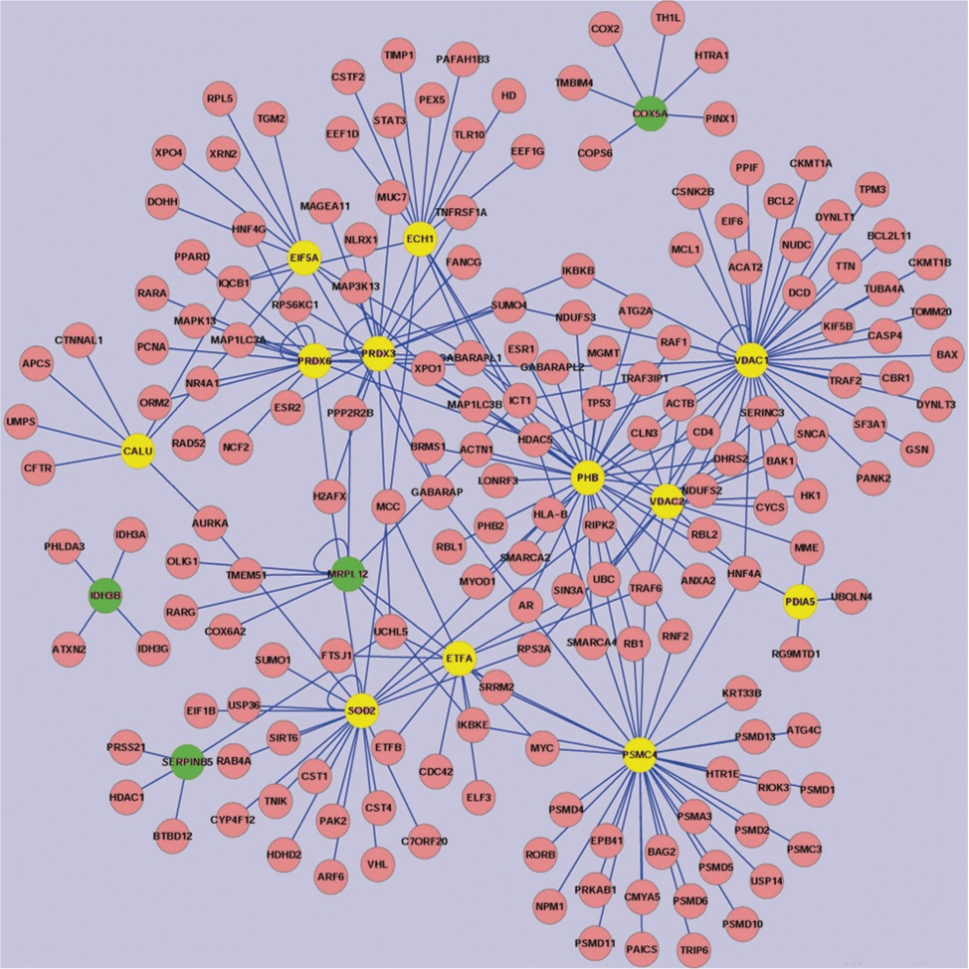
**A protein-protein interaction sub-network of differentially expressed mitochondrial proteins between metastatic 5-8F and nonmetastatic 6-10B cells.** It contains 193 nodes (red, green and yellow circles) and 252 edges (blue lines). A green circle means a down-regulated protein in metastatic 5-8F cells relative to nonmetastatic 6-10B cells. A yellow circle means an up-regulated protein in metastatic 5-8F cells relative to nonmetastatic 6-10B cells.

After data extraction from HPRD and NCBI databases, a protein-protein interaction sub-network of mitochondrial DEPs was generated. These results only extended one layer connection, contained 193 nodes and 252 edges (Figure 5). In this complex sub-network, the colored circles (nodes) represented proteins and the blue full lines (edges) represented protein-protein interactions. The nodes representing 16 DEPs were labeled in yellow (up-regulated) or green (down-regulated). Seven DEPs that interacted more proteins were VDAC1, VDAC2, PRDX3, PRDX6, SOD2, PHB, and PSMC4. These interaction data provided important clues to insight into the molecular mechanisms of NPC metastasis. 

### Validation of PRDX3 expression in mitochondria of metastatic and nonmetastatic cells by Western blotting

**Figure 6:**
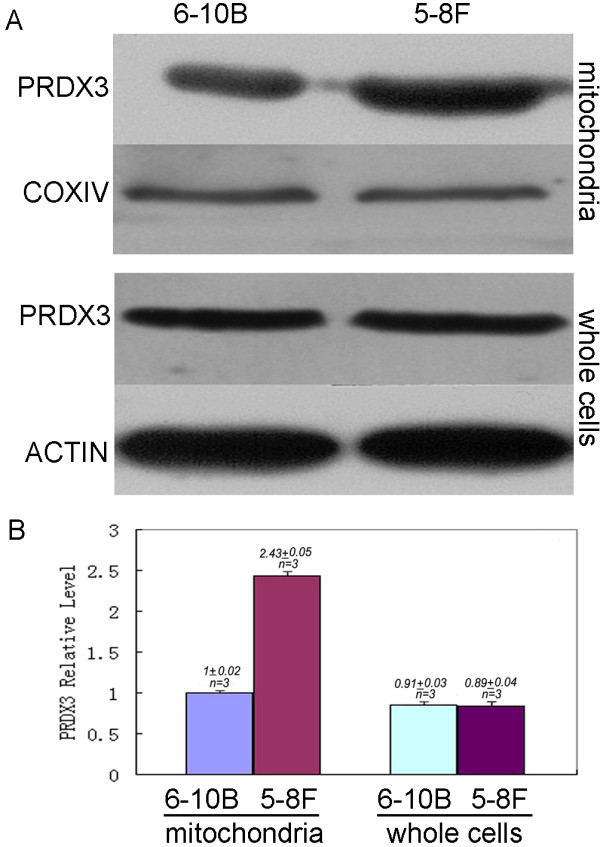
**Up-regulated expressions of PRDX3 in metastatic 5-8F cell mitochondria relative to nonmetastatic 6-10B cell mitochondria without significant expression difference in the level of whole cells.A**. Western blotting image. **B**. The quantitative comparison of the digitized Western blotting image. COXIV, a marker protein of mitochondria, was used as a reference standard protein when mitochondrial protein sample was analyzed with Western blot. ACTIN that is mainly expressed in the cytoplasm was used a reference standard protein when whole-cell protein sample was analyzed with Western blot. Western blot experiments were carried out triply (n = 3).

Western blotting was conducted to confirm differential expression of PRDX3 in mitochondria of metastatic 5-8F cells compared with nonmetastic 6-10B cells. Equal amount (60 μg) of mitochondrial proteins of metastatic 5-8F and nonmetastatic 6-10B cells were applied to 12% SDS-PAGE and then transferred to PVDF membranes using COX IV as internal standard. The results showed that PRDX3 was significantly up-regulated (about 240%) in mitochondria of metastatic 5-8F cells relative to nonmetastatic 6-10B cells (Figure 6). It was consistent with the 2D-DIGE result. 

### Efficiency of PRDX3 siRNA transient transfection in mitochondria of metastatic 5-8F cells

**Figure 7:**
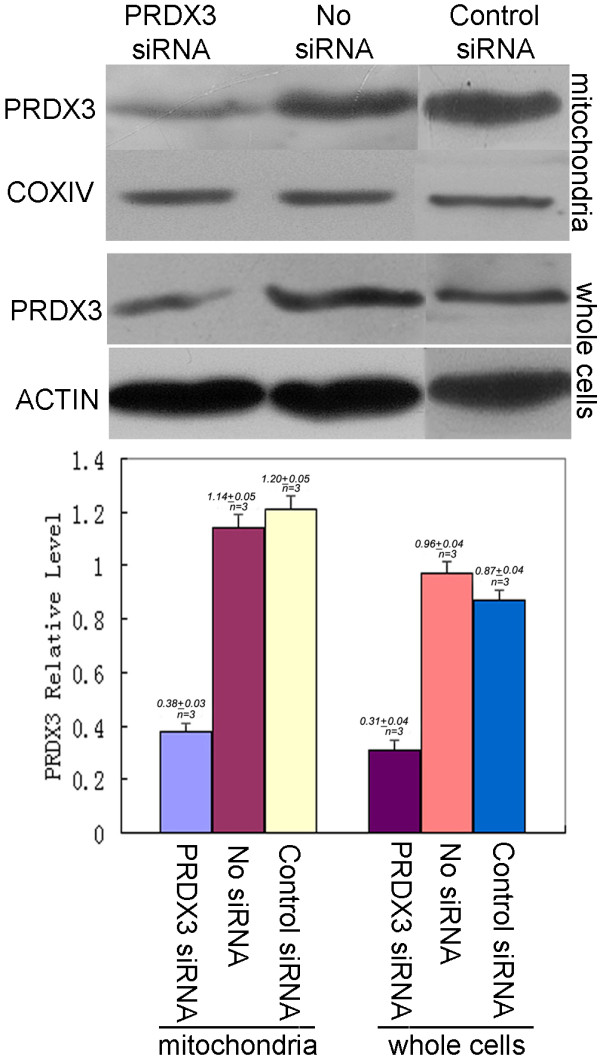
**Effects of PRDX3 siRNA transient transfection on the PRDX3 expression of metastatic 5-8F cells.** Suppression of PRDX3 expression in the levels of mitochondria and whole cells of the metastatic 5-8F cells treated with siRNA relative to without siRNA and control siRNA treatments. The top shows the Western blotting image. The bottom shows the quantitative comparison of the digitized Western blotting image. COXIV, a marker protein of mitochondria, was used as a reference standard protein when mitochondrial protein sample was analyzed with Western blot. ACTIN that is mainly expressed in the cytoplasm was used a reference standard protein when whole-cell protein sample was analyzed with Western blot. Western blot experiments were carried out triply (n = 3).

Efficiency of the PRDX3 siRNA (sc-40833) transient transfection of metastatic 5-8F cells was tested with Western Blotting, using a scrambled sequence siRNA (sc-44230) as control. The result data showed PRDX3 expression in mitochondria of metastatic 5-8F cells with PRDX3 siRNA transient transfection was down-regulated (about 65%) compared with metastatic 5-8F cells without PRDX3 siRNA transient transfection (Figure 7). It demonstrates that expression of PRDX3 was inhibited in the mitochondria of NPC metastatic 5-8F cells with PRDX3 siRNA treatment. 

### Count of metastatic 5-8F cells migrated in Transwell Migration assay

**Figure 8:**
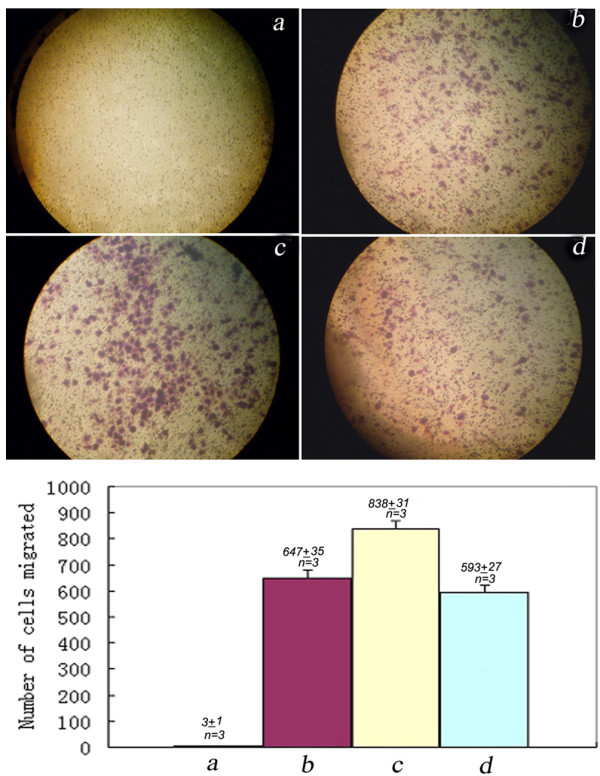
**Effects of PRDX3 siRNA transient transfection on the migration capability of metastatic 5-8F cells.** Effect of PRDX3 siRNA transient transfection on migration capability of metastatic 5-8F cells. The top shows four representative micrographs of metastatic 5-8F cells migrated through Transwell insert membranes among four experimental groups (400 ×). The bottom shows the quantitative comparison of the number of cells that migrated through Transwell insert membranes. Group A (**a**): nonmetastatic 6-10B cells. Group B (**b**): metastatic 5-8F cells. Group C (**c**): metastatic 5-8F cells with PRDX3 siRNA transient transfection. Group D (**d**): metastatic 5-8F cells with a scramble sequence siRNA transient transfection. Transwell migration assays were carried out triply (n = 3).

In three independent experiments, the number of cells migrated through Transwell insert membrane was counted for four experimental groups: Group A (nonmetastatic 6-10B cells; 0 ± 0), Group B (metastatic 5-8F cells; 647 ± 35), Group C (metastatic 5-8F cells with PRDX3 siRNA transient transfection; 838 ± 31), and Group D (metastatic 5-8F cells with a scramble sequence siRNA transient transfection; 593 ± 27). The number of Group C was about 40% more than the number of Group B and Group D (P < 0.05). The effect of PRDX3 siRNA transient transfection on migration capability of metastatic 5-8F cells was showed (Figure 8). It demonstrates that suppression of PRDX3 in metastatic 5-8F cells increased its mobility potential. 

## Discussion

Nasopharyngeal carcinoma (NPC) is a malignant tumor with a high incidence of metastasis, has a great tendency to invade adjacent regions and metastasize to regional lymph node and distant organs. Though the molecular mechanisms of NPC metastasis remain poorly understood, the functions of mitochondria that involved in this process have been uncovered gradually: mitochondria are closely related to oxidative stress process in a cell. Different from the historical views, which assumed oxidative stress contributes to tumor initiation and progression solely by inducing genomic instability, the present views consider oxidative stress directly contributes to tumor metastasis. According to the tumor-stroma co-evolution model, cancer cells induce oxidative stress in adjacent stromal cells and result in an excess production of ROS in mitochondria. Excess stromal ROS induces cytoskeleton rearrangements, autophagy, and mitophagy in the tumor microenvironment to help cancer cells escape excess ROS in the primary tumor site, also induces the stromal over-production of recycled nutrients such as ketones and L-lactate that are fuel for tumor growth and metastasis [10-16]. Moreover, the enhanced cellular antioxidant capability and antioxidant function of redox-signaling in cancer cells result in an increase of cell resistance to oxidant, defend cancer cells themselves from oxidative damage, and favor their metastasis [14].

In general the balance between ROS generation and elimination is delicately maintained in cells. However, for many cancer cells, an increase in ROS production or decrease in ROS-scavenger capacity disturbs redox homeostasis which leads to an overall increase of intracellular ROS level. Over-expressed catalase to alleviate mitochondrial oxidative stress in a mouse animal model of breast cancer could reduce metastatic tumor burden by >12-fold [13]. Considered this catalase experimental result and our PRDX3 study, it is very likely that a molecular network based on redox-signaling pathways in cancer cells directly contributes to tumor metastasis, this process relates closely to mitochondrial oxidative stress and ROS produced by mitochondria might play an important role in tumorigenesis (its direct action on growth factors and transcription factors) and tumor metastasis as well.

**Figure 9:**
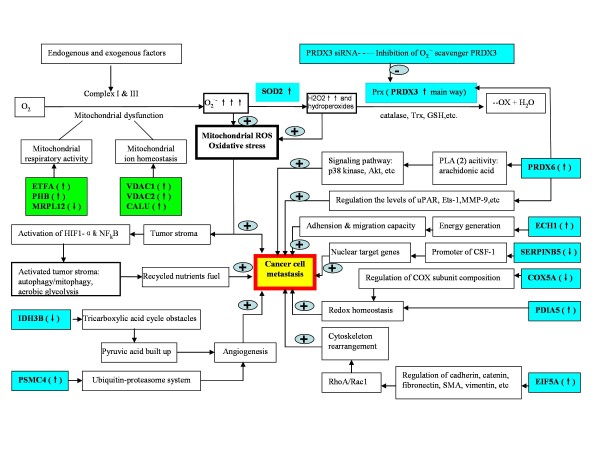
**Experimental data-based diagram that rationalizes differentially expressed mitochondrial proteins in a tumor-stroma co-evolution model.** ↑: up-regulated protein in metastatic 5-8F cells relative to nonmetastatic 6-10B cells. ↓: down-regulated protein in metastatic 5-8F cells relative to nonmetastatic 6-10B cells. The green frame means the relationship between proteins and tumor metastasis is unclear. The blue frame means the relationship between proteins and tumor metastasis is, at least partially, clear. The blue frame with solid line means protein promotes metastasis and the blue frame with dotted line means protein inhibits metastasis. SOD2, superoxide dismutase [Mn] mitochondrial isoform B; PRDX3, peroxiredoxin 3; PRDX6, peroxiredoxin 6; ECH1, mitochondrial delta(3,5)-delta(2,4)-dienoyl-CoA isomerase; SERPINB5, maspin; COX5A, mitochondrial cytochrome c oxidase subunit 5A; PDIA5, protein disulfide; EIF5A, eukaryotic initiation factor 5A; IDH3B, mitochondrial isocitrate dehydrogenase [NAD] subunit alpha; ETFA, electron transfer flavoprotein; PHB, prohibitin; MRPL12, mitochondrial ribosomal protein L7/L12; PSMC4, 26S protease regulatory subunit 7 isoform 1; VDAC1 and VDAC2, voltage-dependent anion channel 1 and 2; and CALU, calumenin isoform c precursor.

Each mitochondrial DEP was rationalized in the tumor-stroma co-evolution model, and an experimental data-based diagram was proposed (Figure 9). In our comparative proteomic study of mitochondria between NPC metastatic 5-8F and nonmetastatic 6-10B cells, the first five enriched GO terms of DEPs were closely related to oxidative stress (Table 2). For PRDX3, our comparative proteomic data demonstrated the expression of PRDX3 in mitochondria of metastatic 5-8F cells is higher than in mitochondria of nonmetastatic 6-10B cells, it shows that the anti-oxidative stress capability in metastatic 5-8F cells is stronger than nonmetastatic 6-10B cells. This result is consistent with previous conclusion [14] that the enhanced anti-oxidant capabilities in cancer cells defend themselves from excess stromal oxidative damage and favors cell metastasis. Furthermore, among several DEPs (data not showed) selected to carry out siRNA transient transfections and then Transwell Migration assay, only suppression of PRDX3 expression resulted in the significantly increased number of cells migrated through Transwell membrane (P < 0.05). PRDX3, mitochondria thioredoxin-dependent peroxide reductase, is the main way by which cancer cells reduce their levels of H_2_O_2_ built up during active respiration [17,18], suppression of this enzyme, a scavenger of peroxide, could increase oxidative stress in cancer cells to up-regulate the redox-signaling, promote the glycolysis, proliferation, and survival, and finally lead increased cells mobility potential [14] (Figure 8). To our knowledge, this was the first experimental evidence that PRDX3 was related to NPC metastasis. 

PRDX6 increased about 2.5-fold in mitochondria of metastatic 5-8F cells than in mitochondria of nonmetastatic 6-10B cells. This result was consistent with the results of other research groups: PRDX6 expressions were increased in node-positive breast carcinoma samples compared with node-negative breast carcinoma samples [19]; enzymatic activities of PRDX6 in lung cancer cells metastasis [20]; over-expression of PRDX6 led to a more invasive phenotype and metastatic potential in human breast cancer [21]. Over-expression of PRDX6 is on the one hand to strengthen the peroxidase activity of a cell to reduce peroxidants such as H_2_O_2_ and hydroperoxides for the relief of oxidative stress. Besides of its peroxidase activity, PRDX6 has additional phospholipase A2 (PLA2) activity, this activity promotes accumulation of arochidonic acid, which, in turn, induces the invasive pathway involving p38 kinase, phosphoinositide 3-kinase, Akt and urokinase-type plasminogen activator. Over-expression of PRDX6 leads to a more invasive phenotype and metastasis potential, at least in part, through regulation of the levels of uPAR, Ets-1, MMP-9, RhoC, and TIMP-2 expression [20,21]. Therefore, PRDX6 in NPC metastatic process demonstrates a double-side effect. To our knowledge this was the first report of the relationship between PRDX6 and NPC metastasis.

SOD2 increased in many tumor types as they progress from early stage non-invasive disease to late stage metastatic disease, and its over-expression in many instances enhance the metastasis phenotype because SOD2 is able to catalize toxic oxidant O_2_^.-^ into another toxic oxidant H_2_O_2_ and hydroperoxides, and O_2_^.-^ and H_2_O_2_ / hydroperoxides both directly contribute to oxidative stress. However, this metastatic phenotype can be reversed by efficient H_2_O_2_ scavenging [22-26]. Our proteomic results also showed that SOD2 increased about 3.3-fold in mitochondria of metastatic 5-8F than in nonmetastatic 6-10B cells. To our knowledge this was the first report of the relationship between SOD2 and NPC metastasis.

Over-expression of ECH1 can enhance the energy generation, and promote adhension and migration capability of cancer cell for cancer metastasis. The present experimental data showed that ECH1 increased in 4.1-fold in mitochondria of NPC metastatic 5-8F than nonmetastatic 6-10B cells. This result was consistent with the other studies [27], a higher expression level of ECH1 was confirmed in tissue from patients with gastric carcinoma with lymph node metastases, and down-regulation of ECH1 could inhibit tumor proliferation, increase the ratio in S phase to G1 phase and decrease the adhesion and migration capacity.

Studies [28] showed over-expression of each SERPINB gene could effectively suppress the invasiveness and motility of malignant cancer cells. Nuclear localization of SERPINB5 is required for its tumor metastasis suppressor function and it was enriched at the promoter of colony-stimulating factor (CSF-1) in chromatin immunoprecipitation and associated with diminished levels of CSF-1 mRNA. It suggests that its mechanism of action involves, in part, direct association of SERPINB5 with target genes to inhibit metastatic phenotype [29]. The present studies showed a down-regulation of SERPINB5 by 3.2-fold in mitochondria of NPC metastatic 5-8F than nonmetastatic 6-10B cells. Thus, the down-regulated SERPINB5 might enhance the metastatic capability of an NPC cancer cell.

COX subunit composition is regulated by COX5A and COX5B gene transcription in response to high and low O_2_, respectively. This subunit switch maintains mitochondria redox homeostasis and relates to morphological and functional alteration of mitochondria [30]. PDIA5 also directly involves in the redox homeostasis. The present studies demonstrate that the down-regulation of COX5A and up-regulation of PDIA5 in NPC metastatic 5-8F cells relative to nonmetastatic 6-10B cells. The abnormal expression of COX5A and PDIA5 in NPC metastatic 5-8F cells might disturb the redox homeostasis to contribute to its metastatic process.

Studies [31] demonstrated over-expression of EIF5A enhances cell motility and promotes tumor metastasis in hepatocellular carcinoma. It was able to induce epithelial-mensenchymal transition (EMT), a key event in tumor invasion and metastasis, characterized by down-regulation of epithelial markers (E-cadherin and beta-catenin) and up-regulation of mesenchymal markers (fibronectin, N-cadherin, alpha-SMA and vimentin). It could also stimulate cytoskeleton rearrangement through activation of RhoA/Rac1 to contribute to the cancer cell metastasis. The present data showed EIF5A increased 2.5-fold in mitochondria of NPC metastatic 5-8F than nonmetastatic 6-10B cells. It suggests that over-expressed EIF5A contributes to NPC cancer cell metastasis.

IDH3B [32] involves in the mitochondrial tricarboxylic acid cycle that is initiated by acetyl-CoA. Acetyl-CoA is derived from the travel of pyruvic acid from cytoplasm into the interior of the mitochondrion. Pyruvic acid is generated from the glycolysis of glucose. The current study demonstrated the down-regulation of mitochondrial IDH3B in NPC metastatic 5-8F cells. Down-regulated IDH3B will obstacle the tricarboxylic acid cycle to reduce the generation of ATP, cause energy metabolism obstacle and mitochondrial dysfunction, and promote oxidative stress. Meanwhile, the tricarboxylic acid cycle obstacle will built up the pyruvic acid. Studies [33] demonstrated that pyruvic acid has strong angiogenic activity and plays an important role in angiogenesis for tumor growth and metastasis.

PSMC4 is a 26S protease that is involved in the ATP-dependent degradation of ubiquitinated proteins in the ubiquitin-proteasome system (UPS) that is extensively associated cancer pathogenesis [34]. The UPS plays a central role in fine-tuning the functions of core proangiogenic proteins including VEGF, VEGFR-2, angiogenic signaling proteins such as the PLCy1 and PI3 kinase/AKT pathways, and other non-VEGF angiogenic pathways to regulate the tumor angiogenesis [35]. The up-regulation of PSMC4 would contribute to this angiogenesis process for tumor metastasis.

Moreover, ETFA is a mitochondrial electron transfer flavoprotein [36]. The ETFA up-regulation in NPC metastatic cell could promote mitochondrial electron transport and oxidative phosphorylation, and enhance mitochondrial oxidative stress. Prohibitin (PHB) is a pro-oncogene, inhibits DNA synthesis, and regulates proliferation. However, PHB gene mutated in human cancers [37], PHB protein responds to mitochondrial stress and may play a role in regulating mitochondrial respiration activity [38]. MRPL12 may act as a scaffolding protein or stabilizer of respiratory chain supercomplexes, is involved in regulation of mitochondrial protein translation and respiration, and plays a role in mitochondrial-mediated death. The current studies found down-regulation of MRPL12, which would regulate mitochondria-mediated cell death and respiration [39,40]. VDAC1 and VDAC2 forms a channel through the mitochondrial outer membrane with a weak anion selectivity at the open state and a cation-selectivity at the close state [41,42], and GALU is involved in the binding of calcium ions [43]. The current studies showed the up-regulated expression of VDAC1, VDAC2, and GALU, they would disturb the mitochondrial ion homeostasis in the mitochondrial dysfunction and oxidative stress. The relationship of those mitochondrial DEPs and the tumor metastasis needs to be validated with experiments; however, these data are novel clues to insight into the mechanisms of NPC metastasis.

## Conclusions

In summary, the present study provides new insights for exploring the significance of mitochondria and oxidative stress in NPC metastatic processes. Ten mitochondrial DEPs including PRDX3, PRDX6, SOD2, ECH1, SERPINB5, COX5A, PDIA5, EIF5A, IDH3B, and PSMC4 have been rationalized in the tumor-stroma co-evolution model that mitochondrial oxidative stress directly contributes to tumor metastasis (Figure 9). The expression suppression of mitochondrial PRDX3 protein in the anti-oxidative stress system enhanced the mobility potential of NPC metastatic cancer cells, which consolidates the roles of mitochondrial oxidative stress in NPC metastasis. Further investigation is required to determine the biological consequences of all identified mitochondrial DEPs and their relevance to the pathogenic mechanisms of NPC metastasis.

## Methods

### Materials

All primary and secondary antibodies, siRNA duplex and a scrambled sequence control siRNA were from Santa Cruz. Lipofectamine^TM^ RNAiMAX transfection reagents and serum-free medium were from Invitrogen. Immobiline DryStrip gels (pH 3-10 NL, 24 cm), CyDye DIGE fluor Cy2, Cy3 and Cy5 were from GE Healthcare. Matrigel basement membrane matrix was from BD Biosciences. Transwell plates and inserts (Cat. No. 3422) were from Corning. All other chemicals, unless stated otherwise, were obtained from Sigma.

### Cells culture

NPC 5-8F and 6-10B cells were obtained from the Modern Analytical Testing Center of Central South University of China. Cells were cultured in RPMI 1640 medium supplemented with 10% fetal bovine serum (FBS) and antibiotics in a humidified incubator with 5% CO_2_ in air at 37°C. The cells were grown to 80% confluence, washed twice with D-Hank’s solution, digested with 0.25% trypsin and 0.02% ethylenediaminetetraacetate dehydrate (EDTA), collected by centrifugation (400 × g, 10 min, 4°C). A total of ~2 × 10^7^ cells were needed for experiments.

### Preparation and characterization of mitochondria, and extraction of mitochondrial proteins

Mitochondria were isolated from 5-8F and 6-10B cells, respectively, according to the Sucrose Gradient Separation Manual (http://www.mitosciences.com) with a minor modification. Briefly, the collected cells described above were resuspended in five volumes of Mito buffers (0.25 M sucrose, 1 mM EDTA, 10 mM Tris·HCl pH 7.4, 1 mM phenylmethanesulfonyl fluoride (PMSF), 1 mg/ml leupeptin, 1 mg/ml pepstatin) and homogenized by pre-cooled Potter-Elvehjem homogenizer (1,000 rpm; around 20 up and down strokes). The homogenates were centrifuged (1,000 × g, 10 min, 4°C) to collect the supernatants. The supernatants were centrifuged (12,000 × g, 15 min, 4°C) to collect the pellets. The pellets were resuspended in 0.5 ml Mito buffers. The discontinuous sucrose gradient separation solutions were prepared in centrifuge tubes with gradient from bottom to top that is 0.5 ml of 2.0 M sucrose, 1 ml of 1.3 M sucrose, 1 ml of 1.0 M sucrose, and 0.5 ml of 0.8 M sucrose. The resuspended samples were carefully applied to the top of gradient solutions and centrifuged (80,000 × g, 2 h, 4°C; Beckman swinging bucket 55 Ti rotors). The brown belts between 1.0 M and 1.3 M sucrose layers were collected carefully with a 5-ml syringe needle immediately after centrifugation. A volume (600 μl) of methanol was added to the top of 150 μl brown sucrose solution, and mixed; then 150 μl of chloroform and 450 μl of water were added, mixed again, and centrifuged (18,000 × g, 5 min, 4°C). All organic and inorganic liquids in centrifuge tubes were poured off carefully, followed by air dry at 4°C of the white discs of proteins. The white discs of proteins were solubilized (2 h; intermittent mix) in 800 μl lysis buffer that contained 8 M urea, 2 M thiourea, 130 mM dithiothreitol (DTT) , 4% 3-[(3-cholanidopropyl) dimethylammonio] -1-propanesulfonate (CHAPS), 40 mM tris base, 1 mM EDTA, 1 mM PMSF, 1 mg/ml leupeptin, 1 mg/ml pepstatin, and pH 8.5 adjusted by 50 mM NaOH, and centrifuged (18,000 × g, 2 h, 4°C). The supernatants were collected. The protein concentration of the supernatants was determined by a 2-D Quantification kit (GE Healthcare). The isolated mitochondria were characterized according to Murayama’s method [44]. Briefly, the extracted mitochondrial proteins were separated by 12% sodium dodecyl sulfate polyacrylamide gel electrophoresis (SDS-PAGE) gels, transferred to polyvinylidene fluoride (PVDF) membranes (Semi-dry Nova blot, Pharmacia Biotech), probed with various primary antibodies against marker proteins from different cellular compartments (LDH, PCNA, COX IV, catalase, GRP 78, and cathepsin D) and corresponding secondary antibodies, and visualized by a SuperSingal West Pico ECL kit (Thermo). Preparation of isolated mitochondria for electron microscopic observation was carried out according to the related manual and observation was made by a Hitachi H 7500 transmission electron microscope.

### 2D-DIGE and imaging analyses

**Figure 10:**
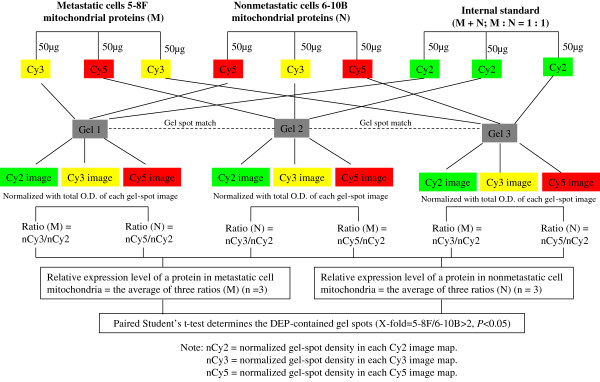
The experimental flow-chart for two-dimensional difference in-gel electrophoresis (2D-DIGE).

The 2D-DIGE experimental flow-chart was shown (Figure 10). Briefly, Mitochondrial proteins were labeled with fluorescent cyanine dyes following the Instruction of Amersham CyDye DIGE Fluors (minimal dyes) for Ettan DIGE Product Booklet. An amount (50 μg) of proteins were labeled with 400 pmol of dyes (dark; on ice; 30 min) and then quenched (10 min) with 1 μl of 10 mM lysine. Two sets of metastatic 5-8F cell proteins were labeled with Cy3 and the third set with Cy5. Two sets of nonmetastatic 6-10B cell proteins were labeled with Cy5 and the third set with Cy3. Each set of above sample contained 50 μg proteins. The internal standard (75 μg of metastatic 5-8F cell proteins plus 75 μg of nonmetastatic 6-10B cell proteins) was labeled with Cy2. One set (50 μg) of Cy3-labeled proteins was combined with one set (50 μg) of Cy5-labeled proteins and combined again with one third (50 μg) of Cy2-labeled internal standard proteins. The combined sample was added in equal volume of 2 × sample buffer (8 M urea, 130 mM DTT, 4% CHAPS, and 2% Pharmalyte 3-10), and then supplied with rehydration buffer (8 M urea, 130 mM DTT, 4% CHAPS, and 1% pharmalyte 3-10) to the total volume of 450 μl. The mixed sample (450 μl) was applied to an immobiline DryStrip gel; and three analytic gels and one preparative gel were simultaneously focused on an IPGphor Unit (GH Healthcare) with a total of 80,000 Vhr. The focused proteins in the immobiline DryStrips were equilibrated in a solution containing 65 mM DTT (15 min), and in another solution containing 135 mM iodacetamide (15 min). The equilibrated proteins in the immobiline DryStrip were separated by 12.5% SDS-PAGE gels in an Ettan DALT II system (GE Healthcare). After electrophoresis, each gel was scanned on a Typhoon 9410 scanner (GE Healthcare) at appropriate wavelengths that were specific for Cy2 (488/520 nm), Cy3 (532/580 nm), and Cy5 (633/670 nm). In total 9 protein-spot image maps were generated. Each gel-spot density was normalized by the total density in each protein-spot image map. The DeCyder differential in-gel analysis (DIA) module was used for pair-wise comparison of each 5-8F and 6-10B protein to the internal standard in each gel. The DeCyder biological variation analysis (BVA) module was then used to simultaneously match all 9 protein-spot image maps. The normalized Cy3/normalized Cy2 and normalized Cy5/normalized Cy2 DIA ratios were used to calculate average abundance changes. The average of three DIA ratios from metastatic cell 5-8F mitochondria represents the relative expression level of a protein in metastatic cell mitochondria, and the average of three DIA ratios from nonmetastatic cell 6-10B mitochondria represents the relative expression level of a protein in nonmetastatic cell mitochondria. The paired Student’s t-test p-values were used for the variance of these ratios for each protein pair across all samples (all 9 protein-spot image maps) to determine each DEP-contained gel-spot. Each DEP-contained gel-spot was excised for protein identification. 

### MS-identification of mitochondrial DEPs

The 2D gel spots that contained DEPs were excised from the preparative gels. The proteins in the gel-spot were destained, reducted, alkylated, and digested in gels with trypsin [45]. For PMF analysis, the tryptic peptides were eluted from ZipTips with 80% acetonitrile/0.1% trifluoroacetic acid, and were spotted onto a MALDI target with an equal volume of cyano-4-hydroxycinnamic acid (10 mg/ml; Sigma) saturated with 50%acetonitrile in 0.05% TFA. The mass spectrometric (MS) spectra were acquired on a Voyager DE STR MALDI-TOF mass spectrometer (ABI, Foster City, CA). The instrument settings were reflector mode with 160-ns delay extraction time, positive polarity, and 20-kV accelerating voltage. The spectrum mass-tocharge (m/z) were usually acquired with laser shots at 200/spectrum and ranged from 1000 to 4000 m/z. External calibration was performed using a Peptide Mass Standard kit (Perspective Biosystems, Framingham, MA). The acquired MS spectra were processed with base-line correction, noise removal (5%), and peak deisotoping using DataExplore (Version 4.0.0.0) software to generate the peaklist for peptide mass fingerprint (PMF) analysis. The PMF data were input into Mascot (http://www.matrixscience.com/) for protein identity searching. The PMF data from the Voyager DE STR mass spectrometer were searched against the NCBInr database (release date January 7, 2012; 16,831,682 sequences, 5,781,564,572 residues; Homo sapiens 241,934 sequences). The search was restricted to “Human” as taxonomy. The other search parameters included type of search (peptide mass fingerprint), enzyme (trypsin), fixed modifications (carbamidomethylcysteine), variable modifications (methionine oxidation), mass values (monoisotopic), protein mass (unrestricted), peptide mass tolerance (100 ppm), peptide charge state (1+), and 1 maximum missed cleavages. Proteins whose scores were greater than 66 were considered as significance (p < 0.05), and only human proteins with the best score in each Mascot search were accepted as successful identifications. The coverage of amino acid sequence, the Mascot score, the mass and pI, and accession number are obtained for each protein.

Proteins in a 2D gel spot that was not identified or was a mixture identified by MALDI-TOF PMF data were subjected to electrospray ionization (ESI)-quadrople (Q)-TOF MS/MS analysis. Briefly, the tryptic peptides from 2D gel spots were loaded onto a C18 pre-column for concentrations and fast desalting, and then eluted to the reversed-phase column for separation. MS/MS spectra were performed in data-depended mode in which up to four precursor ions above an intensity threshold of 7 counts/seconds (cps) were selected for MS/MS analysis from each survey scan. For MS/MS database query, the peptide sequence tag (PKL) format file that was generated from MS/MS data with MassLynx v 4.0 software were inputted into the Mascot search engine to search protein against the NCBInr database (release date January 7, 2012; 16,831,682 sequences, 5,781,564,572 residues; Homo sapiens 241,934 sequences). A mass tolerance of 0.3 Da for both parent (MS) and fragmented (MS/MS) ions, allowance for up to one trypsin miscleavage, variable amino acid modifications consisting of methionine oxidation and cysteine carbamidomethylation were used. MS/MS ion score threshold was determined to produce a false-positive rate less than 5% for a significant hit (P < 0.05). The false-positive rate was calculated with 2∗ reverse/(reverse + forward)/100. In the current study, the MS/MS ion score threshold was 45 and a false-positive rate was approximately 3.1%. Only protein IDH3B was identified with only one peptide with a Mascot score of 73, its MS/MS spectrum was further checked manually and interpreted with de-novo sequencing.

### Functional enrichment analyses of mitochondrial DEPs and protein-protein interaction data

The gene names of mitochondrial DEPs were converted to NCBI-Entrez format for consistency and saved as a text file that was inputted into Cytoscape v 2.8.2 (http://www.cytoscape.org), and BiNGO plugin 2.44 downloaded from Cytoscape manage plugin was used to analyze the enriched biological processes and molecular functions. Human protein-protein interaction data that were downloaded from HPRD (release 9; http://www.hprd.org) and NCBI databases (http://www.ncbi.nlm.nih.gov) were used to generate a protein-protein interaction sub-network of mitochondrial DEPs; the result only extended one layer connection and could be visualized by Cytoscape.

### siRNA transient transfection

The siRNA transient transfections of metastatic 5-8F cells were conducted according to siRNA Transfection Protocols (Santa Cruz) and Transfection Technology Protocols (Invitrogen) with a minor modification. Briefly, the cells were incubated in a six wells plate (Costar) at 37°C in a CO_2_ incubator till 70-80% confluence. The solution A (2.4 μl RNAiMAX in 100 μl serum-free medium) and solution B (10 μl siRNAs duplex in 100 μl serum-free medium) were mixed gently (the final concentration in each well was 100 nM), and incubated (room temperature; 45 min). Cells were washed twice with serum-free medium, and then added into 200 μl of above A + B mixture and 800 μl of serum-free medium in each well. After cells were incubated (7 h), the medium in each well were removed and replaced with 2.5 ml normal fresh growth medium, cells were continued to be incubated (48 h). Cells were washed twice with D-Hank’s solution, digested with 0.25% trypsin and 0.02% EDTA, collected by centrifugation (400 × g; 10 min; 4°C). A scrambled sequence control siRNA experiment was conducted at the same time. The efficiency of siRNA transfection was tested by Western blotting.

### Transwell Migration assays

Cells Transwell Migration assay was carried out according to Matrigel Basement Membrane Matrix Product Specification Sheet (BD Biosciences) and Transwell Insert Product Description (Corning) with a minor modification. Briefly, the Matrigel basement membrane matrix was thawed (4°C; overnight), the Transwell plates was put and inserted (both were added with serum-free medium) in a CO_2_ incubator (37°C; 2 h). The medium in Transwell plates was removed and Transwell plates were inserted and kept on ice. Pre-cooled pipettes were used to add 20 μl Matrigel matrix (1:1 diluted with pre-cooled serum-free medium) into each compartment of Transwell inserts and swirled immediately to disperse materials evenly, placed Transwell inserts (37°C; 30 min) to form thin Matrigel. A volume (600 μl) of medium (RPMI 1640 with 10% FBS and 5 μg/ml fibronectin) was added to each Transwell plates well, followed by adding the above thin Matrigel inserts, then 200 μl medium and the cells (resuspended cells in medium at a density of 5 × 10^4^ cells/ml) were added into each compartment of thin Matrigel inserts, and incubated in a CO_2_ incubator (37°C; 24 h). All of these experiments should be carefully handled using sterile techniques. Those non-migrated cells on each compartment of thin Matrigel inserts were scraped off with cotton swabs, the migrated cells were fixed and stained by 0.1% crystal violet solution. The cells without siRNA transient transfection and with a scrambled sequence transfection were used as control groups. In three independent experiments, the number of cells that migrated through Transwell insert membrane was counted in 9 visual fields per well at 400 × under an Olympus microscope, means of these counts were calculated for statistical analysis using SPSS software, and variables were compared using Student’s t-test with a significance level of P = 0.05.

## Abbreviations

CHAPS: 3-[(3-cholanidopropyl)dimethylammonio]-1-propanesulfonate; DEG: Differentially expressed gene; DEP: Differentially expressed protein; DTT: Dithiothreitol; EDTA: Ethylenediaminetetraacetate dehydrate; ESI: Electrospray ionization; GO: Gene ontology; MALDI: Matrix-assisted laser desorption ionization; MS: Mass spectrometry; MS/MS: Tandem mass spectrometry; NPC: Nasopharyngeal carcinoma; PMF: Peptide mass fingerprint; PMSF: Phenylmethanesulfonyl fluoride; PRDX3: Peroxiredoxin 3; ROS: Reactive oxygen species; 2D-DIGE: Two-dimensional difference in-gel electrophoresis; TOF: Time-of-flight.

## Competing interests

The authors declare no competing interests.

## Authors’ contributions

Conception and design of experiments: JL, XZ and ZC. Development of methodology and acquisition of data: JL, ML, GL, PZ and ZX. Analysis and interpretation of data: JL and XZ. Writing, review, and/or revision of manuscript: JL, XZ and ZC. Administrative, technical, or material support: MS, FP and RH. Study supervision: XZ and ZC. All authors read and approved the final manuscript.

## Pre-publication history

The pre-publication history for this paper can be accessed here:

http://www.biomedcentral.com/1755-8794/5/62/prepub

## Supplementary Material

Additional file 1 **Figure 1.** Protein spot 1. **Figure 2.** Protein spot 2. **Figure 3.** Protein spot 3. **Figure 4.** Protein spot 4. **Figure 5.** Protein spot 5. **Figure 6.** Protein spot 6. **Figure 7.** Protein spot 7. **Figure 8.** Protein spot 8. **Figure 9.** Protein spot 9. **Figure 10.** Protein spot 10. **Figure 11.** Protein spot 11. **Figure 12.** Protein spot 12. **Figure13.** Protein spot 13. **Figure 14.** Protein spot 14. **Figure 15.** Protein spot 15. Click here for file
