# Supplementary material for: Mitochondrial proteomics of nasopharyngeal carcinoma metastasis
Source: BMC Med Genomics. 2012 Dec 6;5:62. doi: 10.1186/1755-8794-5-62 (PMC3539862; doi:10.1186/1755-8794-5-62)
Supplement: Additional file 1 — Figure 1. Protein spot 1. Figure 2. Protein spot 2. Figure 3. Protein spot 3. Figure 4. Protein spot 4. Figure 5. Protein spot 5. Figure 6. Protein spot 6. Figure 7. Protein spot 7. Figure 8. Protein spot 8. Figure 9. Protein spot 9. Figure 10. Protein spot 10. Figure 11. Protein spot 11. Figure 12. Protein spot 12. Figure13. Protein spot 13. Figure 14. Protein spot 14. Figure 15. Protein spot 15. [file 1755-8794-5-62-S1.doc]

**Supplementary Mass Spectral Data**

**Part 1. MALDI-TOF MS and Search Results**

**Sp-Fig.1. Protein spot 1**

**A. MALDI-TOF MS spectrum**


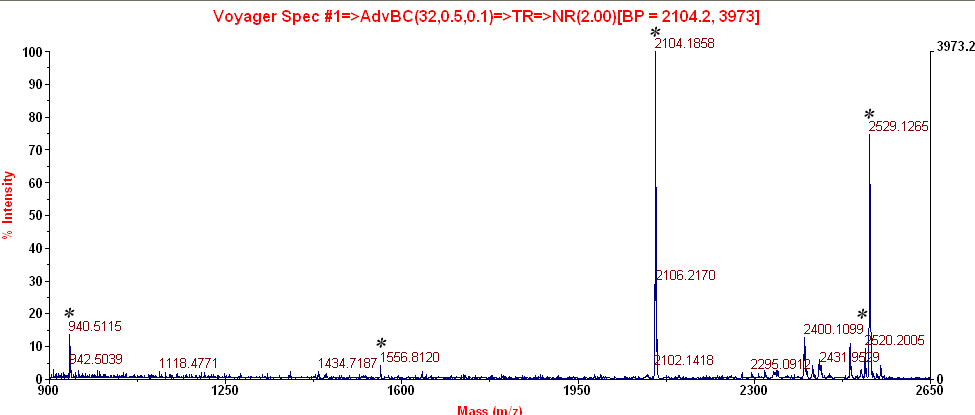


**B.** Peptides detected by MALDI-TOF-MS

| **Peptide** | **Amino acid sequence** | **[M+H]^+^** | **Matched** |
| --- | --- | --- | --- |
| 86-107 | WNTDNTLGTEIAIEDQICQGLK | 2519.1730 | + |
| 108-121 | LTFDTTFSPNTGKK | 1556.8310 | + |
| 178-185 | NNFAVGYR | 940.5110 | + |
| 186-208 | TGDFQLHTNVNDGTEFGGSIYQK | 2528.1460 | + |
| 248-267 | VNNSSLIGVGYTQTLRPGVK | 2103.2000 | + |

**C. Matched peptides (Bold) in the Voltage-dependent anion channel 2 amino acid sequence**

**1** MATHGQTCAR PMCIPPSYAD LGKAARDIFN KGFGFGLVKL DVKTKSCSGV

**51** EFSTSGSSNT DTGKVTGTLE TKYKWCEYGL TFTEK**WNTDN TLGTEIAIED**

**101 QICQGLKLTF DTTFSPNTGK K**SGKIKSSYK RECINLGCDV DFDFAGPAIH

**151** GSAVFGYEGW LAGYQMTFDS AKSKLTR**NNF AVGYRTGDFQ LHTNVNDGTE**

**201 FGGSIYQK**VC EDLDTSVNLA WTSGTNCTRF GIAAKYQLDP TASISAK**VNN**

**251 SSLIGVGYTQ TLRPGVK**LTL SALVDGKSIN AG

**Sp-Fig.2. Protein spot 2**

**A. MALDI-TOF MS spectrum**

**
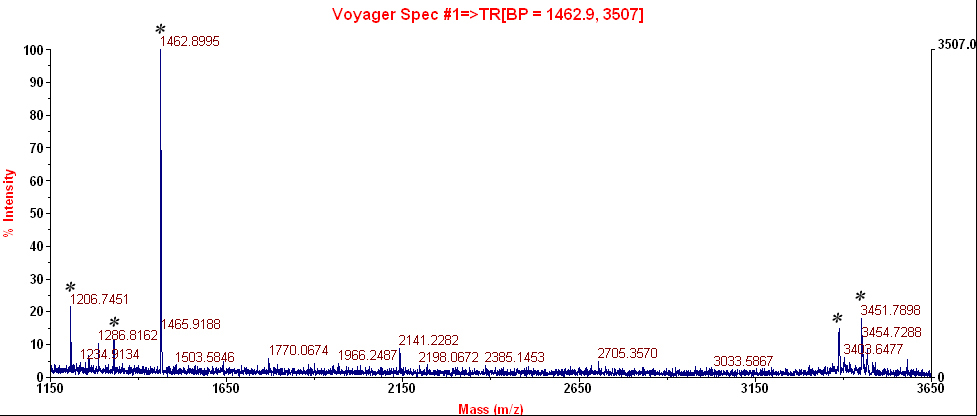
**

**B.** Peptides detected by MALDI-TOF-MS

| **Peptide** | **Amino acid sequence** | **[M+H]^+^** | **Matched** |
| --- | --- | --- | --- |
| 88-117 | ANEFHDVNCEVVAVSVDSHFSHLAWINTPR | 3450.8550 | + |
| 140-153 | DYGVLLEGSGLALR | 1462.9310 | + |
| 154-165 | GLFIIDPNGVIK | 1285.8500 | + |
| 166-176 | HLSVNDLPVGR | 1206.7670 | + |
| 187-217 | AFQYVETHGEVCPANWTPDSPTIKPSPAASK | 3385.8710 | + |

**C. Matched peptides (Bold) in the Peroxiredoxin 3 amino acid sequence**

**1** CVSARPVAPE CTEDGGCCRT VAPSVGSSCH APAVTQHAPY FKGTAVVNGE

**51** FKDLSLDDFK GKYLVLFFYP LDFTFVCPTE IVAFSDK**ANE FHDVNCEVVA**

**101 VSVDSHFSHL AWINTPR**KNG GLGHMNIALL SDLTKQISR**D YGVLLEGSGL**

**151 ALRGLFIIDP NGVIKHLSVN DLPVGR**SVEE TLRLVK**AFQY VETHGEVCPA**

201 NWTPDSPTIK PSPAASKEYF QKVNQ

**Sp-Fig.3. Protein spot 3**

1. **MALDI-TOF MS spectrum**


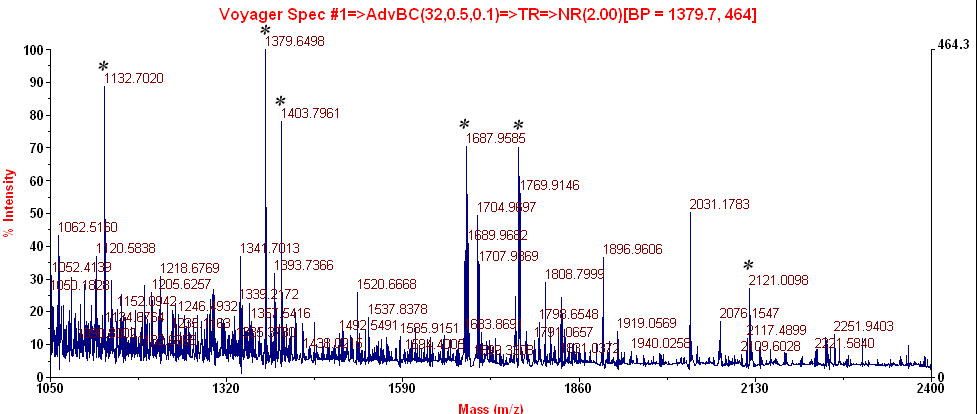


**B.** Peptides detected by MALDI-TOF-MS

| **Peptide** | **Amino acid sequence** | **[M+H]^+^** | **Matched** |
| --- | --- | --- | --- |
| 121-139 | FVVDLSDQVAPTDIEEGMR | 2121.0270 | + |
| 187-200 | LREVVETPLLHPER | 1687.9780 | + |
| 269-284 | ACLIFFDEIDAI GGAR | 1767.9340 | + |
| 285-297 | FDDGAGGDNEVQR | 1379.6370 | + |
| 340-351 | KIEFSLPDLEGR | 1403.7860 | + |
| 367-375 | DIRFELLAR | 1132.7030 | + |

**C. Matched peptides (Bold) in the 26S protease regulatory subunit 7 isoform 1 amino acid sequence**

**1** MPDYLGADQR KTKEDEKDDK PIRALDEGDI ALLKTYGQST YSRQIKQVED

**51** DIQQLLKKIN ELTGIKESDT GLAPPALWDL AADKQTLQSE QPLQVARCTK

**101** IINADSEDPK YIINVKQFAK **FVVDLSDQVA PTDIEEGMR**V GVDRNKYQIH

**151** IPLPPKIDPT VTMMQVEEKP DVTYSDVGGC KEQIEK**LREV VETPLLHPER**

**201** FVNLGIEPPK GVLLFGPPGT GKTLCARAVA NRTDACFIRV IGSELVQKYV

**251** GEGARMVREL FEMARTKK**AC LIFFDEIDAI GGARFDDGAG GDNEVQR**TML

**301** ELINQLDGFD PRGNIKVLMA TNRPDTLDPA LMRPGRLDR**K IEFSLPDLEG**

**351 R**THIFKIHAR SMSVER**DIRF ELLAR**LCPNS TGAEIRSVCT EAGMFAIRAR

**401** RKIATEKDFL EAVNKVIKSY AKFSATPRYM TYN

**Sp-Fig.4. Protein spot 4**

1. **MALDI-TOF MS spectrum**


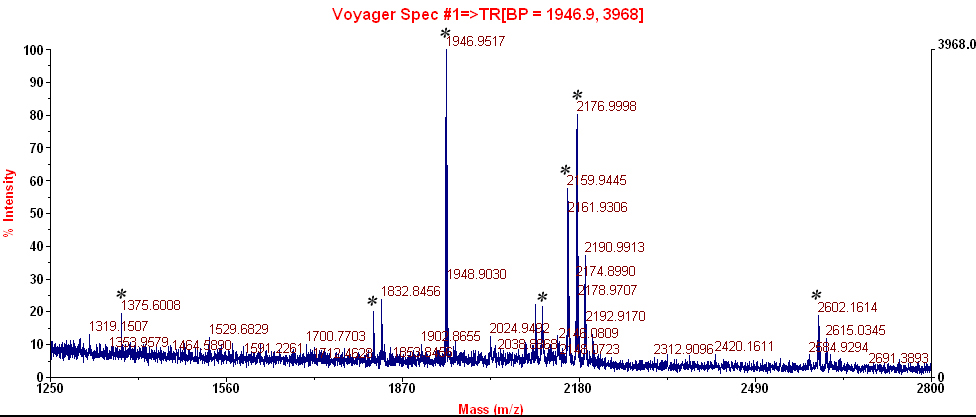


**B.** Peptides detected by MALDI-TOF-MS

| **Peptide** | **Amino acid sequence** | **[M+H]^+^** | **Matched** |
| --- | --- | --- | --- |
| 64-74 | WTEYGLTFTEK | 1374.5980 | + |
| 75-93 | WNTDNTLGTEITVEDQLAR | 2175.9910 | + |
| 121-139 | EHINLGCDMDFDIAGPSIR | 2159.9550 | + |
| 175-197 | TDEFQLHTNVNDGTEFGGSIYQK | 2600.1670 | + |
| 201-218 | KLETAVNLAWTAGNSNTR | 1945.9590 | + |
| 202-218 | LETAVNLAWTAGNSNTR | 1817.8530 | + |
| 237-256 | VNNSSLIGLGYTQTLKPGIK | 2103.1160 | + |

**C. Matched peptides (Bold) in the Volotage-dependent anion-selective channel protein 1 amino acid sequence**

**1** MAVPPTYADL GKSARDVFTK GYGFGLIKLD LKTKSENGLE FTSSGSANTE

**51** TTKVTGSLET KYR**WTEYGLT FTEKWNTDNT LGTEITVEDQ LAR**GLKLTFD

**101** SSFSPNTGKK NAKIKTGYKR **EHINLGCDMD FDIAGPSIR**G ALVLGYEGWL

**151** AGYQMNFETA KSRVTQSNFA VGYK**TDEFQL HTNVNDGTEF GGSIYQK**VNK

**201 KLETAVNLAW TAGNSNTR**FG IAAKYQIDPD ACFSAK**VNNS SLIGLGYTQT**

**251 LKPGIK**LTLS ALLDGKNVNA GGHKLGLGLE FQA

**Sp-Fig.5. Protein spot 5**

1. **MALDI-TOF MS spectrum**

**
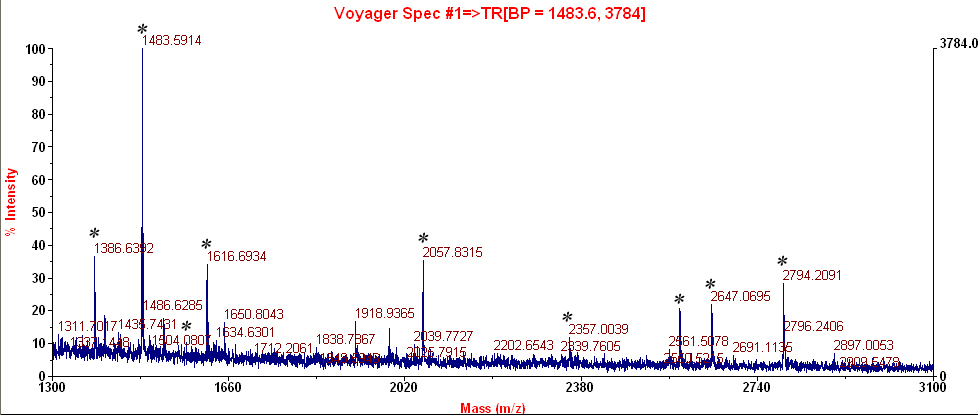
**

**B.** Peptides detected by MALDI-TOF-MS

| **Peptide** | **Amino acid sequence** | **[M+H]^+^** | **Matched** |
| --- | --- | --- | --- |
| 1-18 | LYSSSDDVIELTPSNFNR | 2056.8290 | + |
| 59-83 | VGAVDADKHHSLGGQYGVQGFP TIK | 2581.0800 | + |
| 100-113 | TGEAIVDAALSALR | 1386.6500 | + |
| 198-212 | LAAVDATVNQVLASR | 1527.7120 | + |
| 246-270 | ALDLFSDNAPPPELLEIINEDIAKR | 2793.2010 | + |
| 271-294 | TCEEHQLCVVAVLPHILDTGAAGR | 2646.0710 | + |
| 355-367 | GSFSEQGINEFLR | 1483.6040 | + |
| 374-390 | GSTAPVGGGAFPTIVER | 1615.7170 | + |
| 374-396 | GSTAPVGGGAFPTIVEREPWDGR | 2355.9640 | + |

**C. Matched peptides (Bold) in the Protein disulfide isomerase-related protein 5 amino acid sequence**

**1 LYSSSDDVIE LTPSNFNR**EV IQSDSLWLVE FYAPWCGHCQ RLTPEWKKAA

**51** TALKDVVK**VG AVDADKHHSL GGQYGVQGFP TIK**IFGSNKN RPEDYQGGR**T**

**101 GEAIVDAALS ALR**QLVKDRL GGRSGGYSSG KQGRSDSSSK KDVIELTDDS

**151** FDKNVLDSED VWMVEFYAPW CGHCKNLEPE WAAAASEVKE QTKGRVK**LAA**

**201 VDATVNQVLA SR**YGIRGFPT IKIFQKGESP VDYDGGRTRS DIVSR**ALDLF**

**251 SDNAPPPELL EIINEDIAKR TCEEHQLCVV AVLPHILDTG AAGR**NSYLEV

**301** LLKLADKYKK KMWGWLWTEA GAQSELETAL GIGGFGYPAM AAINARKMKF

**351** ALLK**GSFSEQ GINEFLR**ELS FGR**GSTAPVG GGAFPTIVER EPWDGR**DGEL

**401** PVEDDIDLSD VELDDLGKDE L

**Sp-Fig. 6. Protein spot 6**

1. **MALDI-TOF MS spectrum**

**
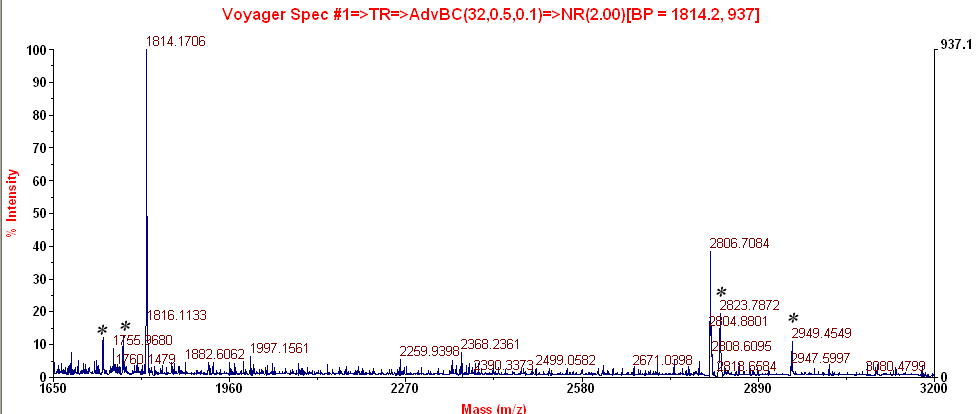
**

**B.** Peptides detected by MALDI-TOF-MS

| **Peptide** | **Amino acid sequence** | **[M+H]^+^** | **Matched** |
| --- | --- | --- | --- |
| 2-28 | QSTLVIAEHANDSLAPITLNTITAATR | 2821.6560 | + |
| 68-83 | GLLPEELTPLILATQK | 1736.1920 | + |
| 84-99 | QFNYTHICAGASAFGK | 1772.0090 | + |
| 277-303 | TIVAINKDPEAPIFQVADYGIVADLFK | 2947.4890 | + |

**C. Matched peptides (Bold) in the Electron transfer flavoprotein amino acid sequence**

**1** M**QSTLVIAEH ANDSLAPITL NTITAATR**LG GEVSCLVAGT KCDKVAQDLC

**51** KVAGIAKVLV AQHDVYK**GLL PEELTPLILA TQKQFNYTHI CAGASAFGK**N

**101** LLPRVAAKLE VAPISDIIAI KSPDTFVRTI YAGNALCTVK CDEKVKVFSV

**151** RGTSFDAAAT SGGSASSEKA SSTSPVEISE WLDQKLTKSD RPELTGAKVV

**201** VSGGRGLKSG ENFKLLYDLA DQLHAAVGAS RAAVDAGFVP NDMQVGQTGK

**251** IVAPELYIAV GISGAIQHLA GMKDSK**TIVA INKDPEAPIF QVADYGIVAD**

**301 LFK**VVPEMTE ILKKK

**Sp-Fig.7. Protein spot 7**

1. **MALDI-TOF MS spectrum**

**
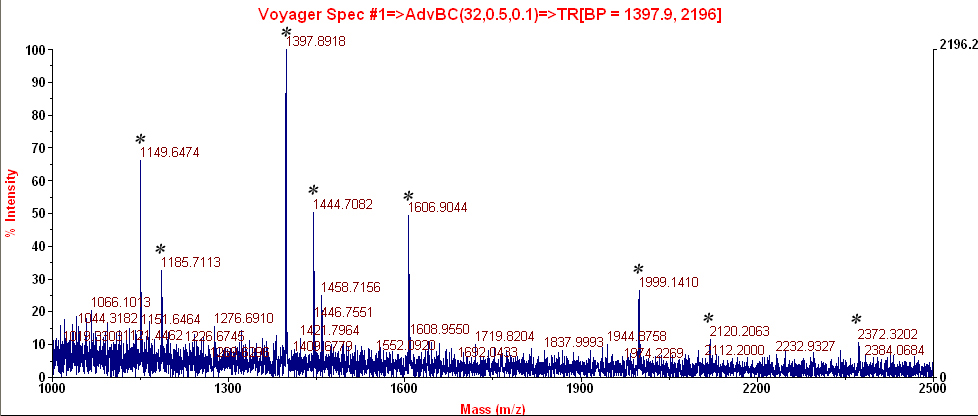
**

**B.** Peptides detected by MALDI-TOF-MS

| **Peptide** | **Amino acid sequence** | **[M+H]^+^** | **Matched** |
| --- | --- | --- | --- |
| 12-35 | FGLALAVAGGVVNSALYNVDAGHR | 2371.3150 | + |
| 84-93 | DLQNVNITLR | 1185.7290 | + |
| 94-105 | ILFRPVASQLPR | 1396.9140 | + |
| 106-117 | IFTSIGEDYDER | 1444.7320 | + |
| 134-143 | FDAGELITQR | 1149.6520 | + |
| 158-177 | AATFGLILDDVSLTHLTFGK | 2119.1780 | + |
| 220-239 | AAELIANSLATAGDGLIELR | 1998.1470 | + |
| 240-253 | KLEAAEDIAYQLSR | 1606.9190 | + |

**C. Matched peptides (Bold) in the Prohibitin amino acid sequence**

**1** MAAKVFESIG K**FGLALAVAG GVVNSALYNV DAGHR**AVIFD RFRGVQDIVV

**51** GEGTHFLIPW VQKPIIFDCR SRPRNVPVIT GSK**DLQNVNI TLRILFRPVA**

**101 SQLPRIFTSI GEDYDER**VLP SITTEILKSV VAR**FDAGELI TQR**ELVSRQV

**151** SDDLTER**AAT FGLILDDVSL THLTFGK**EFT EAVEAKQVAQ QEAERARFVV

**201** EKAEQQKKAA IISAEGDSK**A AELIANSLAT AGDGLIELRK LEAAEDIAYQ**

**251 LSR**SRNITYL PAGQSVLLQL PQ

**Sp-Fig.8. Protein spot 9**

1. **MALDI-TOF MS spectrum**

**
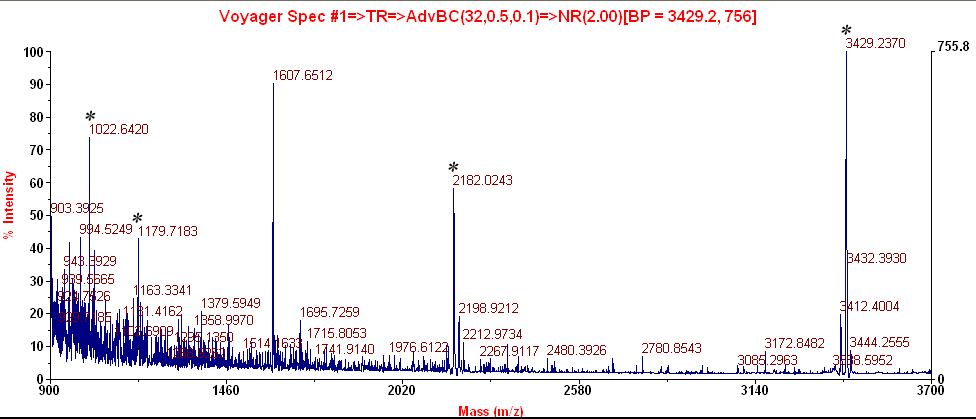
**

**B.** Peptides detected by MALDI-TOF-MS

| **Peptide** | **Amino acid sequence** | **[M+H]^+^** | **Matched** |
| --- | --- | --- | --- |
| 56-72 | WVTYFNKPDIDAWELRK | 2181.0170 | + |
| 98-107 | RLNDFASTVR | 1178.7170 | + |
| 99-107 | LNDFASTVR | 1022.6380 | + |
| 121-150 | EIYPYVIQELRPTLNELGISTPEELGLDKV | 3428.2810 | + |

**C. Matched peptides (Bold) in the Cytochrome c oxidase subunit 5A, mitochondrial precursor amino acid sequence**

**1** MLGAALRRCA VAATTRADPR GLLHSARTPG PAVAIQSVRC YSHGSQETDE

**051** EFDAR**WVTYF NKPDIDAWEL RK**GINTLVTY DMVPEPKIID AALRACR**RLN**

**101 DFASTVR**ILE VVKDKAGPHK **EIYPYVIQEL RPTLNELGIS TPEELGLDKV**

**Sp-Fig.9. Protein spot 10**

1. **MALDI-TOF MS spectrum**

**
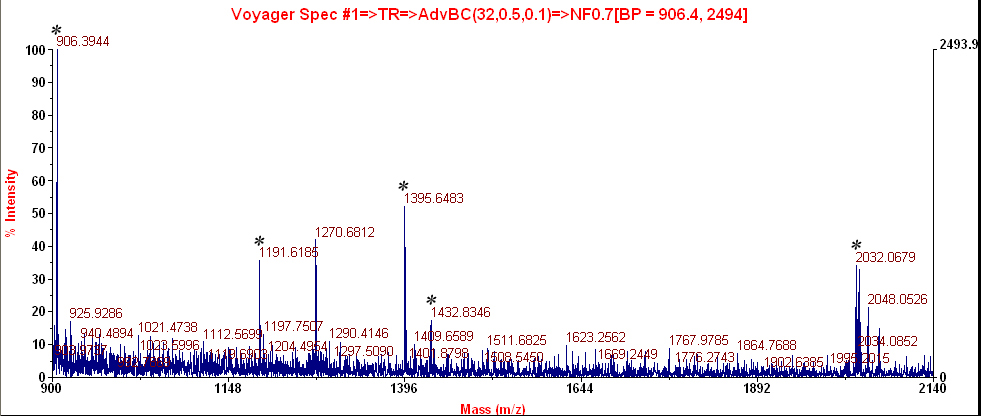
**

**B.** Peptides detected by MALDI-TOF-MS

| **Peptide** | **Amino acid sequence** | **[M+H]^+^** | **Matched** |
| --- | --- | --- | --- |
| 25-41 | FHDFLGDSWGILFSHPR | 2031.0510 | + |
| 42-53 | DFTPVCTTELGR | 1395.6460 | + |
| 143-155 | LKLSILYPATTGR | 1432.8160 | + |
| 145-155 | LSILYPATTGR | 1191.6340 | + |
| 156-162 | NFDEI LR | 906.3950 | + |

**C. Matched peptides (Bold) in the Peroxiredoxin 6 amino acid sequence**

**1** MPGGLLLGDV APNFEANTTV GRIR**FHDFLG DSWGILFSHP RDFTPVCTTE**

**51 LGR**AAKLAPE FAKRNVKLIA LSIDSVEDHL AWSKDINAYN CEEPTEKLPF

**101** PIIDDRNREL AILLGMLDPA EKDEKGMPVT ARVVFVFGPD KK**LKLSILYP**

**151 ATTGRNFDEI LR**VVISLQLT AEKRVATPVD WKDGDSVMVL PTIPEEEAKK

**201** LFPKGVFTKE LPSGKKYLRY TPQP

**Sp-Fig.10. Protein spot 11**

1. **MALDI-TOF MS spectrum**

**
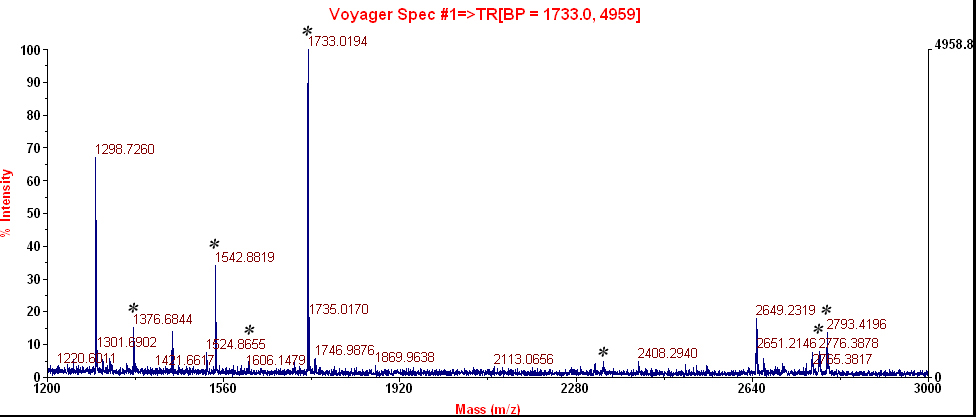
**

**B.** Peptides detected by MALDI-TOF-MS

| **Peptide** | **Amino acid sequence** | **[M+H]^+^** | **Matched** |
| --- | --- | --- | --- |
| 33-59 | LTGSSAQEEASGVALGEAPDHSYESLR | 2761.3280 | + |
| 66-78 | HVLHVQLNRPNKR | 1610.9870 | + |
| 159-185 | CPKPVIAAVHGGCIGGGVDLVTACDIR | 2792.4150 | + |
| 186-196 | YCAQDAFFQVK | 1376.6900 | + |
| 197-211 | EVDVGLAADVGTLQR | 1542.8830 | + |
| 215-230 | VIGNQSLVNELAFTAR | 1732.0200 | + |
| 246-267 | VFPDKEVMLDAALALAAEISSK | 2318.2290 | + |

**C. Matched peptides (Bold) in the Delta(3,5)-Delta(2,4)-dienoyl-CoA isomerase, mitochondrial precursor amino acid sequence**

**1** MAAGIVASRR LRDLLTRRLT GSNYPGLSIS LR**LTGSSAQE EASGVALGEA**

**51 PDHSYESLR**V TSAQK**HVLHV QLNRPNKR**NA MNKVFWREMV ECFNKISRDA

**101** DCRAVVISGA GKMFTAGIDL MDMASDILQP KGDDVARISW YLRDIITRYQ

**151** ETFNVIER**CP KPVIAAVHGG CIGGGVDLVT ACDIRYCAQD AFFQVKEVDV**

**201 GLAADVGTLQ R**LPK**VIGNQS LVNELAFTAR** KMMADEALGS GLVSR**VFPDK**

**251 EVMLDAALAL AAEISSK**SPV AVQSTKVNLL YSRDHSVAES LNYVASWNMS

**301** MLQTQDLVKS VQATTENKEL KTVTFSKL

**Sp-Fig.11. Protein spot 12**

1. **MALDI-TOF MS spectrum**

**
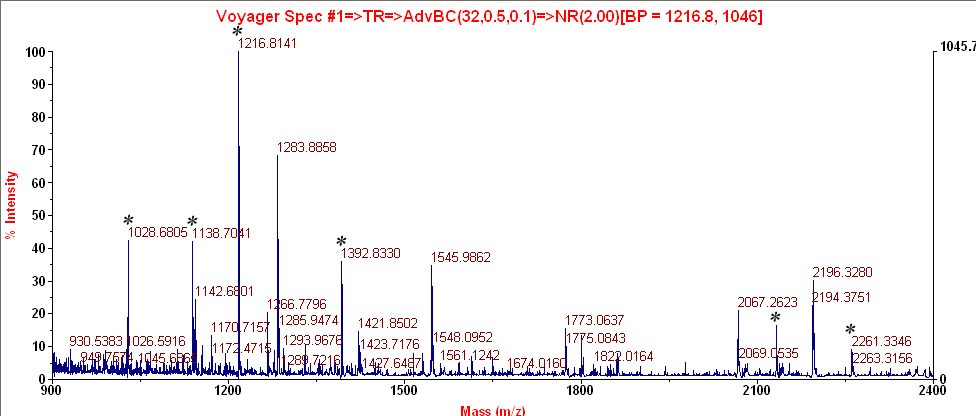
**

**B.** Peptides detected by MALDI-TOF-MS

| **Peptide** | **Amino acid sequence** | **[M+H]^+^** | **Matched** |
| --- | --- | --- | --- |
| 59-66 | APIQWEER | 1028.5320 | + |
| 116-134 | KTFDLYANVR | 2260.1460 | + |
| 117-134 | TFDLYANVR | 2132.0630 | + |
| 135-146 | PCVSIEGYKTPYTDVNIVTIR | 1391.7840 | + |
| 179-188 | IAEFAFEYAR | 1216.6170 | + |
| 215-223 | CREVAESCK | 1138.5740 | + |

**C. Matched peptides (Bold) in the Isocitrate dehydrogenase [NAD] subunit alpha, mitochondrial precursor amino acid sequence**

**1** MAGPAWISKV SRLLGAFHNP KQVTRGFTGG VQTVTLIPGD GIGPEISAAV

**51** MKIFDAAK**AP IQWEER**NVTA IQGPGGKWMI PSEAKESMDK NKMGLKGPLK

**101** TPIAAGHPSM NLLLR**KTFDL YANVRPCVSI EGYKTPYTDV NIVTIR**ENTE

**151** GEYSGIEHVI VDGVVQSIKL ITEGASKR**IA EFAFEYAR**NN HRSNVTAVHK

**201** ANIMRMSDGL FLQK**CREVAE SCK**DIKFNEM YLDTVCLNMV QDPSQFDVLV

**251** MPNLYGDILS DLCAGLIGGL GVTPSGNIGA NGVAIFESVH GTAPDIAGKD

**301** MANPTALLLS AVMMLRHMGL FDHAARIEAA CFATIKDGKS LTKDLGGNAK

**351** CSDFTEEICR RVKDLD

**Sp-Fig.12. Protein spot 13**

1. **MALDI-TOF MS spectrum**

**
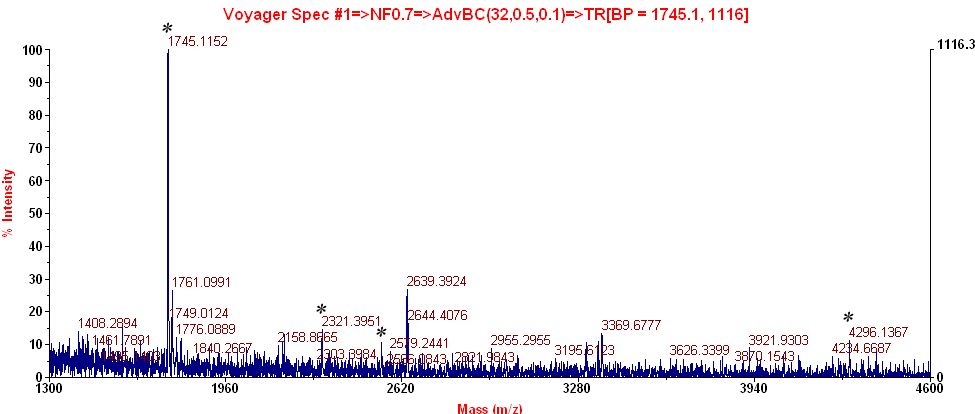
**

**B.** Peptides detected by MALDI-TOF-MS

| **Peptide** | **Amino acid sequence** | **[M+H]^+^** | **Matched** |
| --- | --- | --- | --- |
| 54-75 | HHAAYVNNLNVTEEKYQEALAK | 2542.4100 | + |
| 96-117 | LTAASVGVQGSGWGWLGFNKER | 2320.3840 | + |
| 118-155 | GHLQIAACPNQDPLQGTTGLIPL GIDVWEHAYYLQYK | 4294.0260 | + |
| 164-177 | AIWNVINWENVTER | 1744.1120 | + |

**C. Matched peptides (Bold) in the Superoxide dismutase [Mn], mitochondrial isoform B precursor amino acid sequence**

**1** MLSRAVCGTS RQLAPVLGYL GSRQKHSLPD LPYDYGALEP HINAQIMQLH

**51** HSK**HHAAYVN NLNVTEEKYQ EALAK**GELLE AIKRDFGSFD KFKEK**LTAAS**

**101 VGVQGSGWGW LGFNKERGHL QIAACPNQDP LQGTTGLIPL LGIDVWEHAY**

**151 YLQYK**NVRPD YLK**AIWNVIN WENVTER**YMA CKK

**Sp-Fig.13. Protein spot 16**

1. **MALDI-TOF MS spectrum**

**
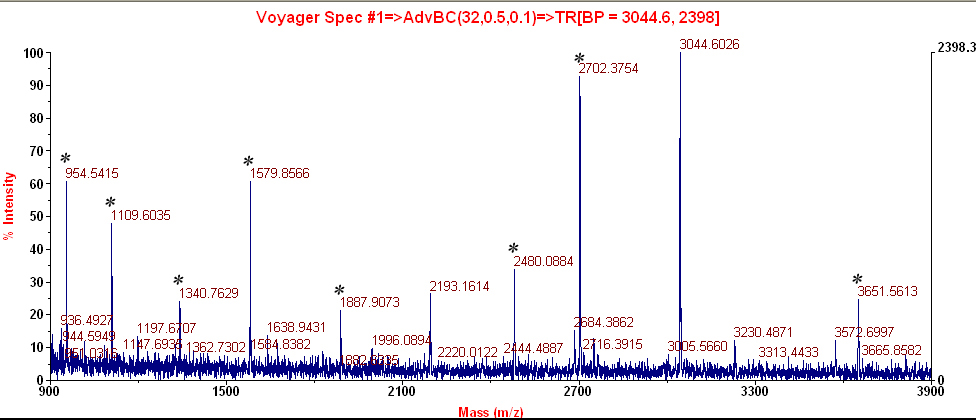
**

**B.** Peptides detected by MALDI-TOF-MS

| **Peptide** | **Amino acid sequence** | **[M+H]^+^** | **Matched** |
| --- | --- | --- | --- |
| 36-67 | VHHEPQLSDKVHNDAQSFDYDHDAFLGAEEAK | 3649.5920 | + |
| 46-67 | VHNDAQSFDYDHDAFLGAEEAK | 2479.1240 | + |
| 68-80 | TFDQLTPEESKER | 1579.8570 | + |
| 112-119 | WIYEDVER | 1109.6190 | + |
| 240-249 | TEREQFVEFR | 1340.7500 | + |
| 243-249 | EQFVEFR | 954.5430 | + |
| 264-279 | DWILPSDYDHAEAEAR | 1887.9360 | + |
| 296-319 | EEIVDKYDLFVGSQATDFGEALVR | 2701.4020 | + |

**C. Matched peptides (Bold) in the Calumenin isoform c precursor amino acid sequence**

**1** MKETDLIIMD LRQFLMCLSL CTAFALSKPT EKKDR**VHHEP QLSDKVHNDA**

**51 QSFDYDHDAF LGAEEAKTFD QLTPEESKER** LGKIVSKIDG DKDGFVTVDE

**101** LKDWIKFAQK R**WIYEDVER**Q WKGHDLNEDG LVSWEEYKNA TYGYVLDDPD

**151** PDDGFNYKQM MVRDERRFKM ADKDGDLIAT KEEFTAFLHP EEYDYMKDIV

**201** VQETMEDIDK NADGFIDLEE YIGDMYSHDG NTDEPEWVK**T EREQFVEFR**D

**251** KNRDGKMDKE ETK**DWILPSD YDHAEAEAR**H LVYESDQNKD GKLTK**EEIVD**

301 KYDLFVGSQA TDFGEALVRH DEF

**Part 2. ESI-Q-TOF MS/MS and Search Results**

**Sp-Fig.14. Protein spot 6**

**A. ESI-Q-TOF MS/MS spectrum of peptide (LGGEVSCLVAGTK m/z 654.7138)**

**
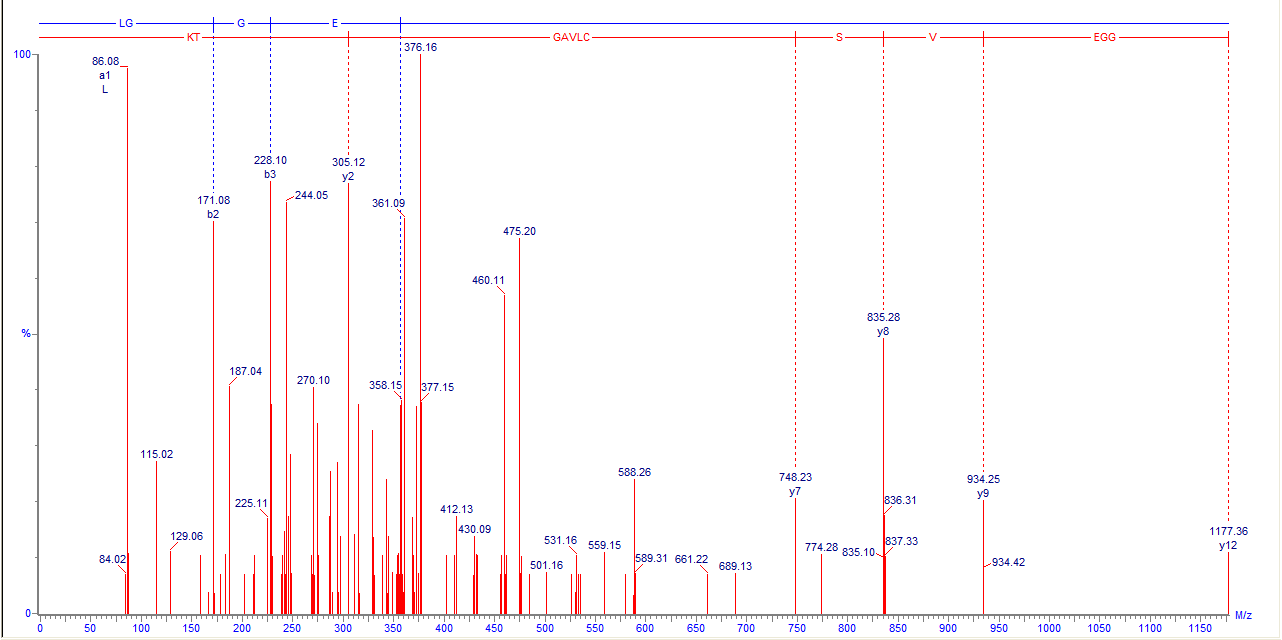
**

**m/z**

B. Peptides characterized by ESI-Q-TOF MS/MS

| **Peptide** | **Amino acid sequence** | **m/z** | **Ion charge** |
| --- | --- | --- | --- |
| 29-41 | LGGEVSCLVAGTK | 654.7138 | 2+ |
| 109-121 | LEVAPISDIIAIK | 691.2985 | 2+ |
| 304-313 | VVPEMTEILK | 579.7211 | 2+ |

1. **Characterized peptides (Bold) in the ETFA amino acid sequence**

**1** MQSTLVIAEH ANDSLAPITL NTITAATR**LG GEVSCLVAGT K**CDKVAQDLC

**51** KVAGIAKVLV AQHDVYKGLL PEELTPLILA TQKQFNYTHI CAGASAFGKN

**101** LLPRVAAK**LE VAPISDIIAI K** SPDTFVRTI YAGNALCTVK CDEKVKVFSV

**151** RGTSFDAAAT SGGSASSEKA SSTSPVEISE WLDQKLTKSD RPELTGAKVV

**201** VSGGRGLKSG ENFKLLYDLA DQLHAAVGAS RAAVDAGFVP NDMQVGQTGK

**251** IVAPELYIAV GISGAIQHLA GMKDSKTIVA INKDPEAPIF QVADYGIVAD

**301** LFK**VVPEMTE ILK**KK

**Sp-Fig.15. Protein spot 8**

**A. ESI-Q-TOF MS/MS spectrum of peptide (KLVESLPQEIK m/z 642.1907)**

**
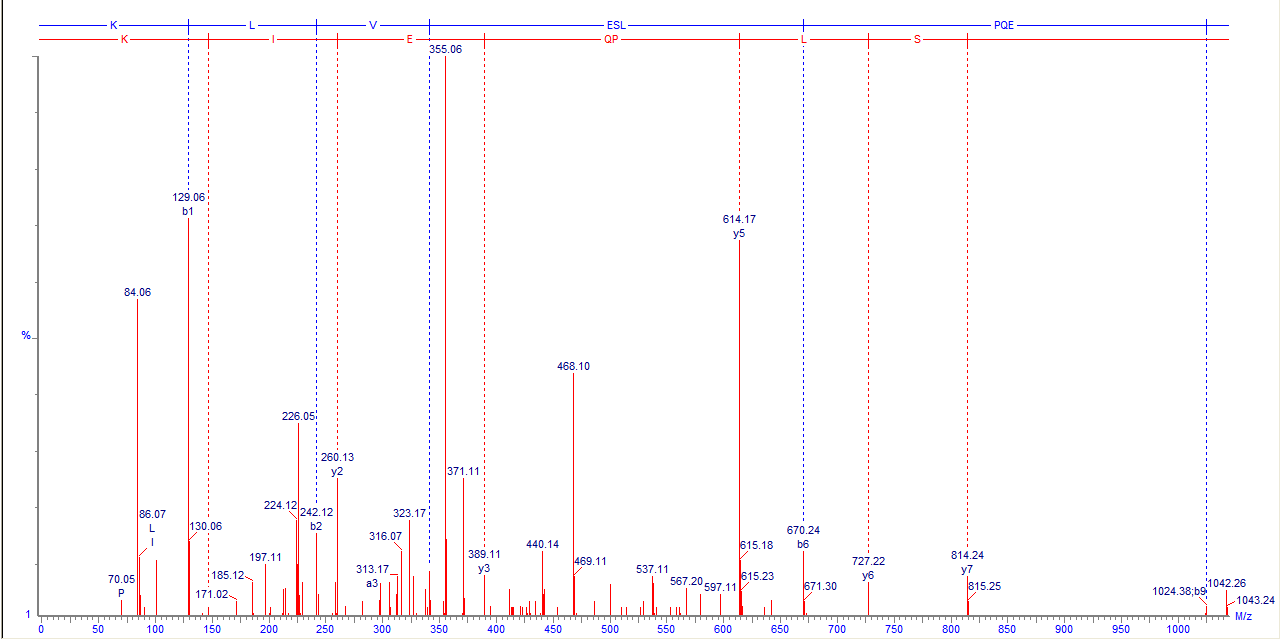
**

**m/z**

**B. Peptides characterized by ESI-Q-TOF MS/MS**

| **Peptide** | **Amino acid sequence** | **m/z** | **Ion charge** |
| --- | --- | --- | --- |
| 151-162 | NYIQGINLVQAK | 680.6882 | 2+ |
| 163-173 | KLVESLPQEIK | 642.1907 | 2+ |
| 184-198 | IKAALEAVGGTVVLE | 735.2183 | 2+ |
| 186-198 | AALEAVGGTVVLE | 614.6719 | 2+ |

**C. Characterized peptides (Bold) in the MRPL12 amino acid sequence**

**1** MLPAAARPLW GPCLGLRAAA FRLARRQVPC VCTVRHMRSS GHQRCEALAG

**51** APLDNAPKEY PPKIQQLVQD IASLTLLEIS DLNELLKKTL KIQDVGLVPM

**101** GGVMSGAVPA AAAQEAVEED IPIAKERTHF TVRLTEAKPV DKVKLIKEIK

**151 NYIQGINLVQ AKKLVESLPQ EIK**ANVAKAE AEK**IKAALEA VGGTVVLE**

**Sp-Fig.16. Protein spot 12**

**A. ESI-Q-TOF MS/MS spectrum of peptide (TPYTDVNIVTIR m/z 696.1862)**

**
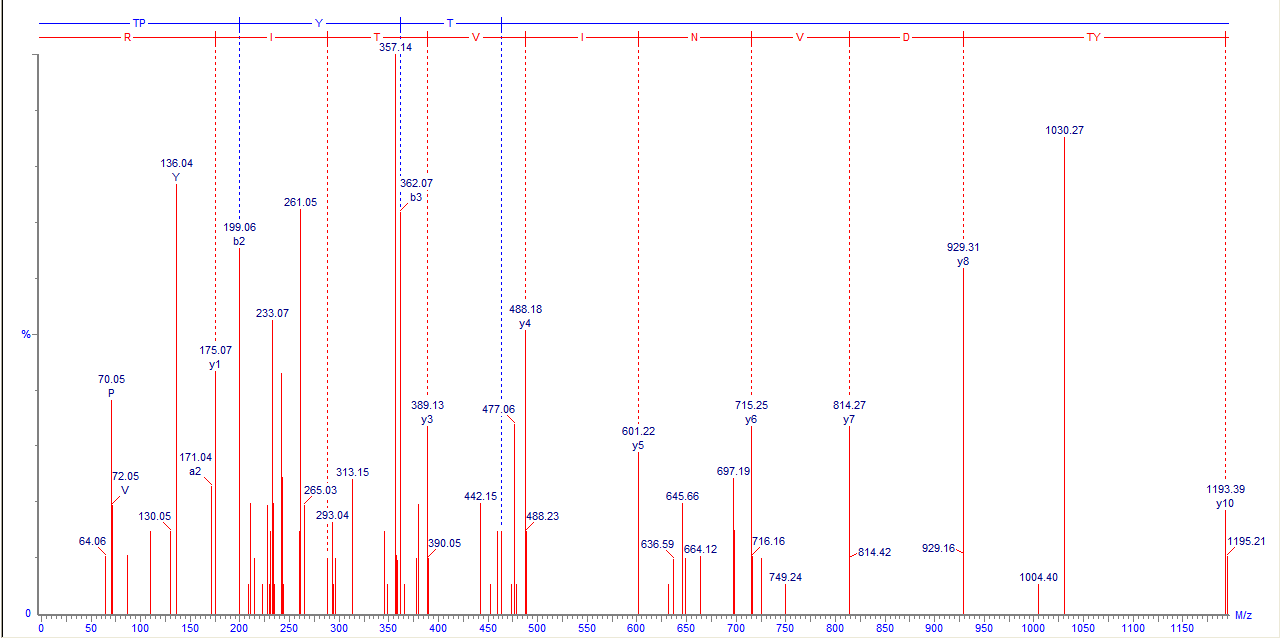
**

**m/z**

**B. Peptides characterized by ESI-Q-TOF MS/MS**

| **Peptide** | **Amino acid sequence** | **m/z** | **Ion charge** |
| --- | --- | --- | --- |
| 135-146 | TPYTDVNIVTIR | 696.1862 | 2+ |

**C. Characterized peptides (Bold) in the IDH3B amino acid sequence**

**1** MAGPAWISKV SRLLGAFHNP KQVTRGFTGG VQTVTLIPGD GIGPEISAAV

**51** MKIFDAAKAP IQWEERNVTA IQGPGGKWMI PSEAKESMDK NKMGLKGPLK

**101** TPIAAGHPSM NLLLRKTFDL YANVRPCVSI EGYK**TPYTDV NIVTIR**ENTE

**151** GEYSGIEHVI VDGVVQSIKL ITEGASKRIA EFAFEYARNN HRSNVTAVHK

**201** ANIMRMSDGL FLQKCREVAE SCKDIKFNEM YLDTVCLNMV QDPSQFDVLV

**251** MPNLYGDILS DLCAGLIGGL GVTPSGNIGA NGVAIFESVH GTAPDIAGKD

**301** MANPTALLLS AVMMLRHMGL FDHAARIEAA CFATIKDGKS LTKDLGGNAK

**351** CSDFTEEICR RVKDLD

**Sp-Fig.17. Protein spot 13**

**A. ESI-Q-TOF MS/MS spectrum of peptide (GELLEAIKR m/z 514.6976)**

**
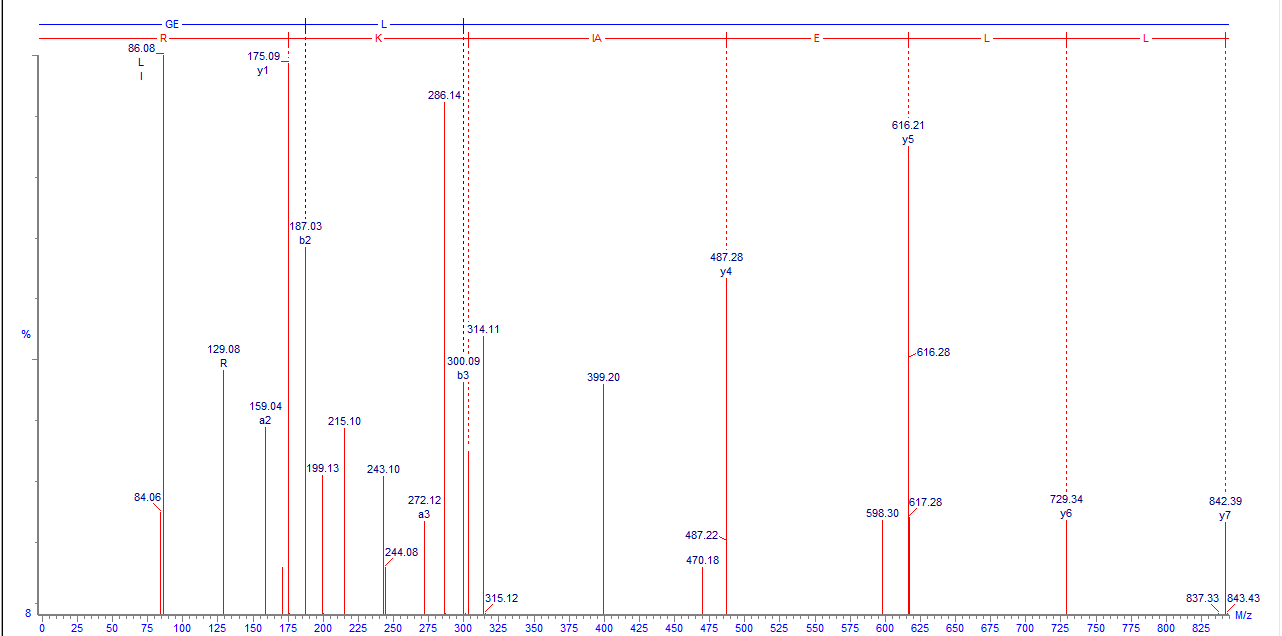
**

**m/z**

B. Peptides characterized by ESI-Q-TOF MS/MS

| **Peptide** | **Amino acid sequence** | **m/z** | **Ion charge** |
| --- | --- | --- | --- |
| 115- 123 | GELLEAIKR | 514.6976 | 2+ |
| 124-132 | DFGSFDKFK | 545.6549 | 2+ |

**C. Characterized peptides (Bold) in the SOD2 amino acid sequence**

**1** MLSRAVCGTS RQLAPALGYL GSRQKHSLPD LPYDYGALEP HINAQIMQLH

**51** HSKHHAAYVN NLNVTEEKYQ EALAKGDVTA QTALQPALKF NGGGHINHSI

**101** FWTNLSPNGG GEPK**GELLEA IKRDFGSFDK FK**EKLTAASV GVQGSGWGWL

**151** GFNKERGHLQ IAACPNQDPL QGTTGLIPLL GIDVWEHAYY LQYKNVRPDY

**201** LKAIWNVINW ENVTERYMAC KK

**Sp-Fig.18. Protein spot 14**

**A. ESI-Q-TOF MS/MS spectrum of peptide (VHLVGIDIFTGK m/z 649.6807)**

**
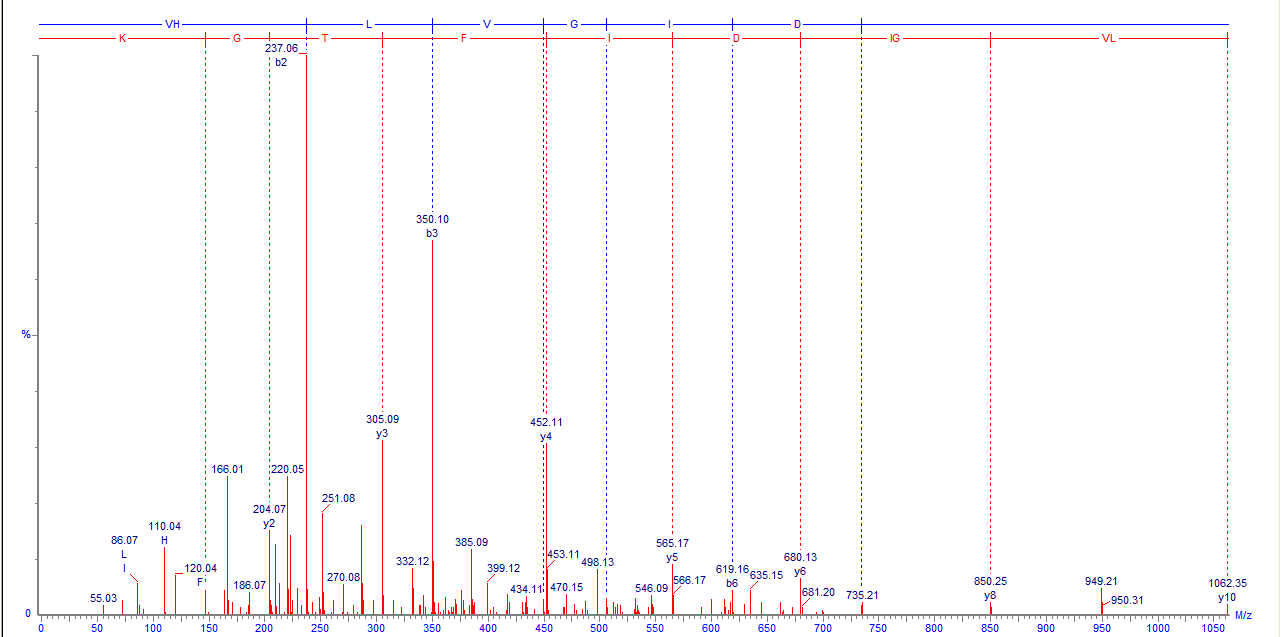
**

**m/z**

B. Peptides characterized by ESI-Q-TOF MS/MS

| **Peptide** | **Amino acid sequence** | **m/z** | **Ion charge** |
| --- | --- | --- | --- |
| 56-67 | VHLVGIDIFTGK | 649.6575 | 2+ |
| 56-67 | VHLVGIDIFTGK | 649.6807 | 2+ |
| 56-68 | VHLVGIDIFTGKK | 713.7011 | 2+ |

**C. Characterized peptides (Bold) in the EIF5A amino acid sequence**

**1** MADDLDFETG DAGASATFPM QCSALRKNGF VVLKGWPCKI VEMSASKTGK

**51** HGHAK**VHLVG IDIFTGKK**YE DICPSTHNMD VPNIRRNDFQ LIGIQDGYLS

**101** LLQDSGEVPE DLRLPEGDLG KEIEQKYDCG EEILITVLSA MTEEAAVAIK

**151** AMAK

**Sp-Fig.19. Protein spot 15**

**A. ESI-Q-TOF MS/MS spectrum of peptide (DVPFGFQTVTSDVNK m/z 827.1690)**

**
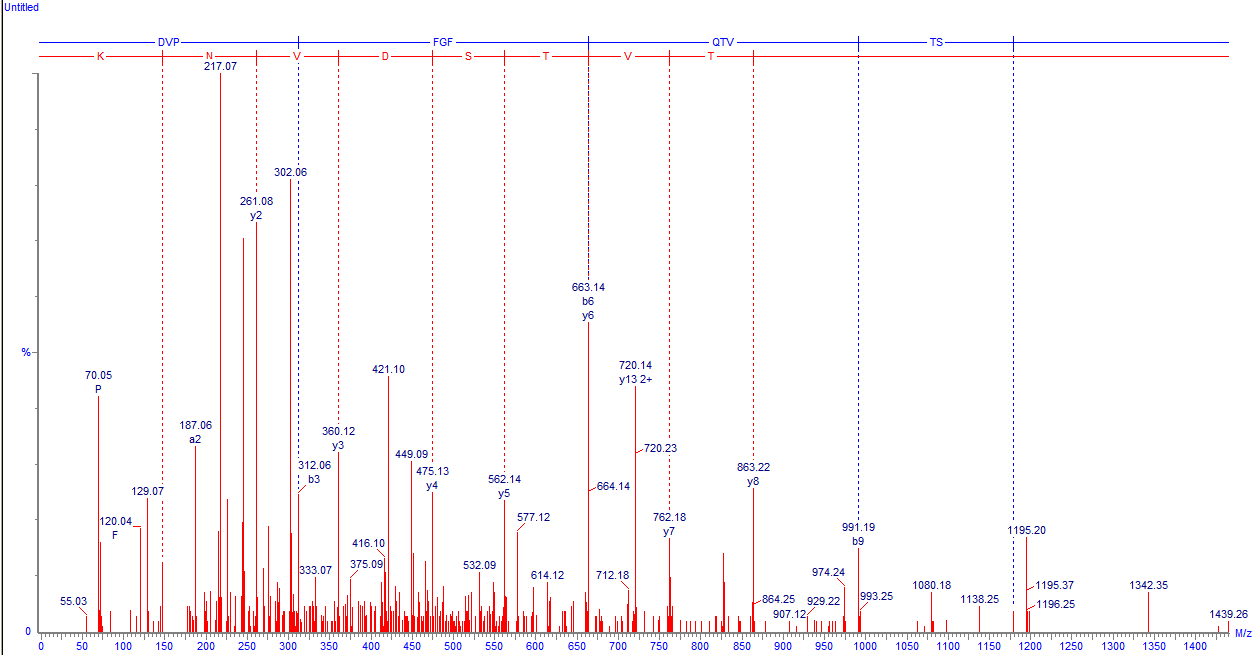
**

**m/z**

B. Peptides characterized by ESI-Q-TOF MS/MS

| **Peptide** | **Amino acid sequence** | **m/z** | **Ion charge** |
| --- | --- | --- | --- |
| 79 - 93 | DVPFGFQTVTSDVNK | 827.1690 | 2+ |
| 111- 123 | SLNLSTEFISSTK | 713.6774 | 2+ |
| 173- 184 | ILVVNAAYFVGK | 647.1919 | 2+ |
| 230- 238 | IIELPFQNK | 551.1615 | 2+ |
| 239- 248 | HLSMFILLPK | 599.6807 | 2+ |

**C. Characterized peptides (Bold) in the Maspin amino acid sequence**

**1** HHHHHHENLY FQGSMDALQL ANSAFAVDLF KQLSEKEPLG NVLFSPIALS

**51** TSLSLAQVGA KGDTANEIGQ VLHFENVK**DV PFGFQTVTSD VNK**LSSFYSL

**101** KLIKRLYVDK **SLNLSTEFIS STK**RPYAKEL ETVDFKDKLE ETKGQINNSI

**151** KDLTDGHFEN ILADNSVNDQ TK**ILVVNAAY FVGK**WMKKFS ESETKESPFR

**201** VNKTDTKPVQ MMNMEATFSM GNIDSINSK**I IELPFQNKHL SMFILLPK**DV

**251** EDESTGLEKI EKQLNSESLS QWTNPSTMAN AKVKLSIPKF KVEKMIDPKA

**301** SLENLGLKHI FSEDTSDFSG MSETKGVALS NVIHKVSLEI TEDGGDSIEV

**351** PGARILQHKD ELNADHPFIY IIRHNKTRNI IFFGKFSSP
